# N^6^-Methyladenosine Modification Profile in Bovine Mammary Epithelial Cells Treated with Heat-Inactivated *Staphylococcus aureus*

**DOI:** 10.1155/2022/1704172

**Published:** 2022-02-23

**Authors:** Ting Li, Yifan Zhu, Changjie Lin, Jie Chen, Yiya Yin, Xin Tang, Yingyu Chen, Aizhen Guo, Changmin Hu

**Affiliations:** ^1^Department of Clinical Veterinary Medicine, Faculty of Veterinary Medicine, Huazhong Agricultural University, Wuhan, 430070 Hubei Province, China; ^2^Department of Preventive Veterinary Medicine, Faculty of Veterinary Medicine, Huazhong Agricultural University, Wuhan, 430070 Hubei Province, China; ^3^State Key Laboratory of Agricultural Microbiology, Huazhong Agricultural University, Wuhan, 430070 Hubei Province, China; ^4^Institute of Animal Husbandry and Veterinary Medicine, Wuhan Academy of Agricultural Sciences, Wuhan, 430023 Hubei Province, China

## Abstract

The symptoms of mastitis caused by *Staphylococcus aureus* (*S. aureus*) in dairy cows are not obvious and difficult to identify, resulting in major economic losses. N^6^-Methyladenosine (m^6^A) modification has been reported to be closely associated with the occurrence of many diseases. However, only a few reports have described the role of m^6^A modification in *S. aureus*-induced mastitis. In this study, after 24 h of treatment with inactivated *S. aureus*, MAC-T cells (an immortalized bovine mammary epithelial cell line) showed increased expression levels of the inflammatory factors IL-1*β*, IL-6, TNF-*α*, and reactive oxygen species. We found that the mRNA levels of METLL3, METLL14, WTAP, and ALKBH5 were also upregulated. Methylated RNA immunoprecipitation sequencing analysis revealed that 133 genes were m^6^A hypermethylated, and 711 genes were m^6^A hypomethylated. Biological functional analysis revealed that the differential m^6^A methylated genes were mainly related to oxidative stress, lipid metabolism, inflammatory response, and so on. In the present study, we also identified 62 genes with significant changes in m^6^A modification and mRNA expression levels. These findings elucidated the m^6^A modification spectrum induced by *S. aureus* in MAC-T cells and provide the basis for subsequent m^6^A research on mastitis.

## 1. Introduction

Mastitis is one of the most common diseases in dairy cows, with a high incidence rate, and its treatment remains a challenge. Most cases of mastitis are caused by pathogenic microorganisms that invade the mammary gland tissues [1]. To date, more than a hundred pathogenic microorganisms have been isolated from bovine mammary gland tissues [2]. When the gland is stimulated by pathogenic microorganisms, bovine mammary epithelial cells activate the innate immune response to resist invasion. Innate immunity plays a major role in the early stages of infection. Pathogen-associated molecular patterns are recognized by receptors on the surface of bovine mammary epithelial cells, causing an immune response that wipes out pathogenic microorganisms [3]. Among the many pathogenic microorganisms that cause mastitis, *Staphylococcus aureus* is one of the most common gram-positive bacteria [1, 4]. Previous studies have shown that *S. aureus* is involved in many pathological processes, including breast tissue destruction and chronic inflammation [5, 6]. The main toxins produced by *S. aureus*, such as endotoxins, and modifications to their peptidoglycan layer and lipoteichoic acid moieties, can cause mastitis [7]. Exotoxins secreted by *S. aureus* can destroy the basic structure of the mammary gland, leading to the degeneration and necrosis of mammary epithelial cells. After *S. aureus* infection, bovine mammary epithelial cells release inflammatory factors through the toll-like receptors (TLRs) and nuclear factor-kappa B (NF-*κ*B) signaling pathway and activate innate immunity [8–10]. In addition, *S. aureus* has been reported to induce fibrosis [11]. Mastitis caused by *S. aureus* infection is usually recessive or subacute; thus, it is not easily detected in clinical settings. Owing to the high infection rate and drug resistance of *S. aureus*, *S. aureus*-induced mastitis is difficult to cure [7, 12]. Therefore, further studies on the mechanism of *S. aureus*-induced mastitis are of great significance for clinical diagnosis, treatment, and prevention.

N^6^-Methyladenosine (m^6^A) modification is a type of RNA modification during which the N^6^ position of adenine in RNA is methylated. As a reversible dynamic modification [13, 14], its biological function is mainly determined by the methylase system [15]. The system includes “Writer,” methyltransferases, including methyltransferase-like 3 (METTL3) and 14 (METTL14), and Wilms tumor 1-associated protein (WTAP) [16, 17]; “Eraser,” demethylase such as fat mass and obesity-associated gene (FTO) and alkB homolog 5 (ALKBH5) [17]; and “Reader,” m^6^A methylated reading proteins [18, 19], such as the YTH family of proteins, IGF2BP protein, and eukaryotic initiation factor (elF3 protein) [20]. Studies have shown that most diseases are accompanied by changes in the levels of methylating enzymes; methylation enzymes are often involved in the regulation of diseases [21–23]. Changes in m^6^A modifications are closely related to immune regulation [24]. Feng et al. found that m^6^A levels are responsible for lipopolysaccharide-induced inflammatory reactions in human dental pulp cells [25]. In addition, m^6^A can also regulate T-cell homeostasis, resistance to viruses and bacteria, and antitumor immunity [26–28]. Furthermore, Jiaxing et al. summarized the role of m^6^A modification in stem cell death and survival and further explained the role of m^6^A modification in immune response, cell apoptosis, autophagy, and senescence [29]. Moreover, m^6^A modification has been confirmed to play an important role in the development of many diseases. However, whether m^6^A affects *S. aureus*-induced mastitis in bovines has not been reported.

In this study, we detected changes in the expression levels of methyltransferase and demethylase in bovine mammary epithelial cells induced by *S. aureus* and analyzed the detected differential m^6^A modified transcripts by using methylated RNA immunoprecipitation sequencing (MeRIP-seq). These findings shed light on the role of m^6^A modification in *S. aureus*-induced mastitis.

## 2. Materials and Methods

### 2.1. Bacterial Strains and Cell Line

MAC-T cells (an immortalized bovine mammary epithelial cell line) were kindly donated by Professor Mark Hanigan of Virginia Tech University. We used DME/F12 medium (Hyclone, Tauranga, New Zealand) supplemented with 10% fetal bovine serum (FBS) (Gibco, New York, NY, United States) to culture the cells, with incubation at 37°C in a 5% CO_2_ incubator. We used 0.25% trypsin and 0.02% EDTA to digest and passage the cells.

*Staphylococcus aureus* (ATCC 29213) was donated by Professor Zhou Rui of Huazhong Agricultural University. A 100 *μ*L *S. aureus* suspension was inoculated into 10 mL Luria-Bertani (LB) liquid medium at a ratio of 1 : 100 and grown in a shaker incubator at 220 rpm/min and 37°C. Then, 100 *μ*L of the bacterial culture was serially diluted from 10^−1^ to 10^−8^, spread plated onto LB solid medium, and incubated overnight in a bacterial incubator at 37°C. The number of bacteria was counted, and the remaining bacteria in the broth culture were inactivated in a water bath at 63°C for 30 min.

### 2.2. Sample Collection and RNA Extraction

The MAC-T cells were seeded at a density of 10^6^ cells per well in a cell culture dish (Corning, New York, NY, USA). The cells were set up in triplicate per group. After 12 h, the DMEM was replaced with 2% FBS in the cell culture dish. Then, the inactive bacterial cells were added to the dish at a 10 : 1 ratio (bacteria: cells) [30]. In the control group, an equal volume of LB medium was added, and the cells were incubated for 24 h. After incubation, we discarded the medium and then used cold phosphate-buffered saline (PBS) to wash the cells three times.

Total RNA was extracted from the MAC-T cells using TRIzol Reagent (Invitrogen, Carlsbad, CA, USA) in accordance with the manufacturer's instructions. The RNA concentrations were measured using a NanoDrop 2000 instrument (Thermo, Waltham, MA, USA). Ribonucleic acid integrity and gDNA contamination were assessed using agarose gel electrophoresis.

### 2.3. Real-Time Quantitative Polymerase Chain Reaction (RT-qPCR)

The RNA samples were reverse transcribed using HiScript III Reverse Transcriptase (Vazyme, Nanjing, Jiangsu, China). Then, we used AceQ qPCR SYBR Green Master Mix (Vazyme) to configure the sample to be tested by RT-qPCR. The samples were placed in a ViiA™ 7 Real-Time PCR System instrument (Applied Biosystems Inc., Foster, CA, USA), and the qPCR program was run. Data were analyzed using the 2^-*ΔΔ*Ct^ method. The relevant primer information is summarized in Table 1.

### 2.4. Enzyme-Linked Immunosorbent Assay (ELISA)

The supernatants of the MAC-T cells exposed to the bacteria for 24 h and of the control group were collected. The concentrations of the inflammatory factors (IL-1*β*, IL-6, and TNF-*α*) in the supernatant were then measured using an ELISA kit (Cusabio, Wuhan, Hubei, China) following the manufacturer's instructions.

### 2.5. Cellular Reactive Oxygen Species Detection

Cellular reactive oxygen species (ROS) were detected using a commercially available kit (Beyotime, Shanghai, China) according to the manufacturer's instructions. Specifically, the supernatant of the MAC-T cells was discarded, and the cells were washed three times with cold PBS. Next, trypsin without EDTA was used to collect the cells. The cells were then resuspended in PBS. Finally, flow cytometry (Beckman Coulter, Indianapolis, IN, USA) was used to detect the fluorescence intensity at 488 nm and 525 nm, respectively.

### 2.6. Methylated RNA Immunoprecipitation Quantitative Polymerase Chain Reaction (MeRIP-qPCR)

The following experiments were performed in accordance with the manufacturer's instructions (Millipore, Bedford, MA, USA): (1) total RNA was fragmented by Zn^2+^ at 94°C; (2) magnetic beads (Thermo Fisher Scientific, Waltham, MA, USA) and m^6^A antibody (Abcam, Cambridge, UK) were incubated for 1 h at room temperature; (3) the system was incubated with the fragmented RNA at 4°C for 2 h; (4) elution buffer was used to elute the mixture twice at 4°C for 1 h; and (5) the collected eluate was subjected to RNA extraction and reverse transcription (Vazyme). In accordance with manufacturer's the instructions, cDNA was detected by RT-qPCR using the AceQ SYBR qPCR Master Mix (Vazyme). The data were analyzed by % input; that is, %input = 2^−(Average CT_RIP_ − Average CT_input_ − log_2_^(input dilution factor)^). CT_RIP_ means the CT value of the RNA immunoprecipitation (IP RNA) samples, and CT_input_ means the CT value of the input RNA samples. The primers for the relevant methylated RNA were as follows (Table 2).

### 2.7. MeRIP-seq and mRNA-seq

The collected RNA was sent for MeRIP-seq and ribonucleic acid sequencing (RNA-seq) at Cloud-Seq Biotech (Shanghai, China) (GSE161050). In this study, the m^6^A-MeRIP kit (Millipore, Burlington, MA, USA) was used to perform the m^6^A RNA immunoprecipitation reaction. RNA sequencing libraries were constructed from the input RNA samples and IP RNA samples after immunoprecipitation with the NEBNext Ultra II Directional RNA Library Prep Kit (New England Biolabs, Ipswich, MA, USA). After library quality control, high throughput sequencing was performed with Illumina HiSeq (Illumina, San Diego, CA, USA).

### 2.8. Bioinformatic Analysis

Clean reads of high quality were obtained after Q30 quality control and removal of the connector using the Cutadapt (v1.9.3 software). Then, HISAT2 (v2.0.4 software) was used to match the clean reads of the samples to the reference genome (bosTau9), and the MACS (v1.4.2 software) was used to identify RNA m^6^A methylation. Enrichment analyses were performed using Gene Ontology (GO, http://www.geneontology.org) and the Kyoto Encyclopedia of Genes and Genomes (KEGG, http://www.genome.jp/kegg) for the differentially methylated genes.

### 2.9. Statistical Analysis

In this study, Prism v7.0 (GraphPad software) was mainly performed using for data analyses. The results are presented as the mean values (±SD) of three independent experiments, and *p* values <0.05 were considered statistically significant.

## 3. Results

### 3.1. Heat-Inactivated *S. aureus* Induced Inflammation and Oxidative Stress in the MAC-T Cells

**Figure 1:**
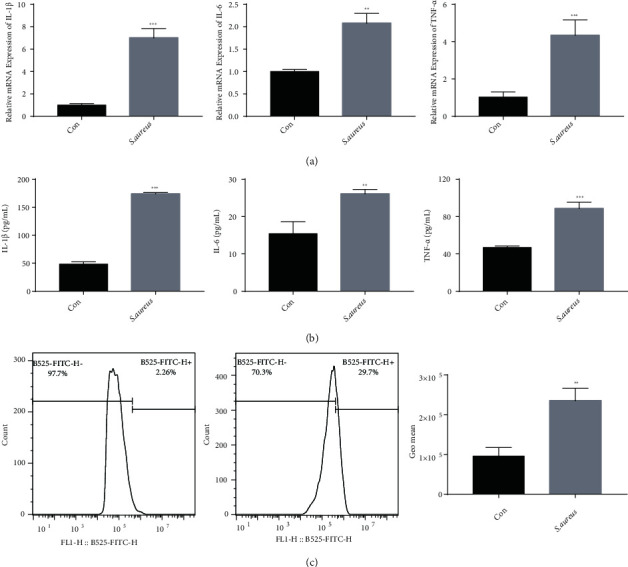
Detection of inflammatory factors and reactive oxygen species in MAC-T cells treated with *S. aureus*. (a) mRNA expressions of the inflammatory factors IL-1*β*, IL-6, and TNF-*α* detected by RT-qPCR. (b) Protein expressions of the inflammatory factors IL-1*β*, IL-6, and TNF-*α* detected by ELISA. (c) Changes in the expression levels of reactive oxygen species in the control and *S. aureus* groups, detected by flow cytometry. ^∗^*p* < 0.05, ^∗∗^*p* < 0.01, and ^∗∗∗^*p* < 0.001.

After the MAC-T cells were stimulated with heat inactivated *S. aureus* at an MOI ratio of 10 : 1 for 24 h, the expression of inflammatory factors was detected using RT-qPCR and ELISA. Compared with the control group, the *S. aureus* group showed significantly increased mRNA and protein levels of IL-1*β*, IL-6, and TNF-*α* (Figures 1(a) and 1(b)). In previous studies, mastitis was also accompanied by oxidative stress [31]. As shown in Figure 1(c), compared with the control group, the expression level of ROS in the *S. aureus* group was significantly increased. These data thus indicated that heat-inactivated *S. aureus* induces inflammation and oxidative stress in MAC-T cells.

### 3.2. Abnormal Expression Levels of m^6^A Transferase/Demethylase Were Induced by *S. aureus* in the MAC-T Cells

**Figure 2:**
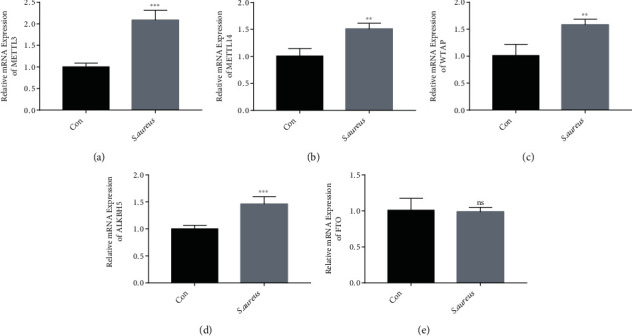
Variation in the mRNA expression levels of the m^6^A enzymes in *S. aureus*-induced mastitis. The mRNA expressions of the methyltransferases METTL3 (a), METTL14 (b), and WTAP (c) and the demethylases ALKBH5 (d) and FTO (e) were detected by RT-qPCR. ns: not significant, ^∗^*p* < 0.01 and ^∗∗^*p* < 0.001.

The m^6^A enzyme system plays an important role in RNA m^6^A modification [18, 19]. Some studies have indicated that the occurrence of disease is related to the abnormal expression of the m^6^A enzymes [32]. In this study, the expression levels of methyltransferases METTL3, METTL14, and WTAP and demethylases ALKBH5 and FTO were detected using RT-qPCR. The results showed that compared with the control group, the *S. aureus* group had significantly increased mRNA expression levels of METTL3, METTL14, WTAP, and ALKBH5 (Figures 2(a)–2(d)), but no significant difference in the expression level of FTO was observed (Figure 2(e)). This suggests that m^6^A modification may be related to the inflammatory response and oxidative stress induced by *S. aureus* in the MAC-T cells.

### 3.3. Overview of the m^6^A Methylation Map in the Control and *S. aureus* Groups

**Figure 3:**
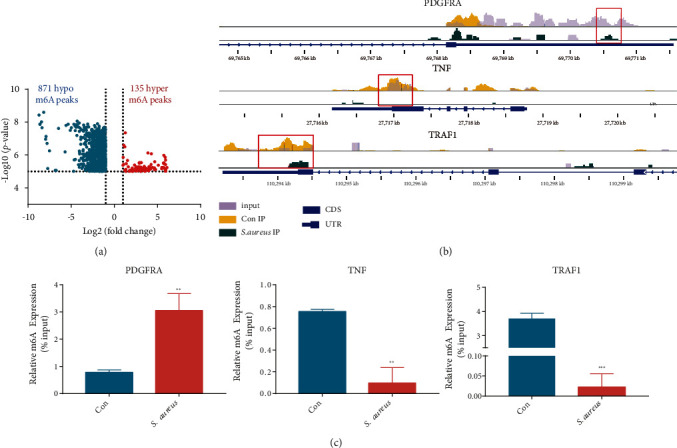
Analysis of differential m^6^A-modified genes between the control and *S. aureus* groups. (a) Volcano plot analysis of differential m^6^A methylation genes (*p* < 0.00001, fold change > 2). (b) Visualization of PDGFRA, TNF, and TRAF1 using the IGV software. (c) Verification of PDGFRA, TNF, and TRAF1 by MeRIP-seq.

Based on the expression levels of the m^6^A enzymes, we speculated that m^6^A methylation differed between the control and the *S. aureus* groups, which was further detected using MeRIP-seq. Compared with the control group, the *S. aureus* group obtained 1,006 significantly distinct m^6^A peaks in 844 mRNAs (*p* < 0.00001, fold change > 2, Table S1), among which 133 mRNAs had 135 hypermethylated sites such as PDGFRA and 711 mRNAs had 871 hypomethylated sites such as TNF and TRAF1 (Figures 3(a) and 3(b)). In addition, by MeRIP-qPCR, this study confirmed the hypermethylation of PDGFRA and hypomethylation of TNF and TRAF1 in *S. aureus*-induced MAC-T cells by MeRIP-qPCR (Figure 3(c)).  Table 3 lists the top 20 differential m^6^A peaks.

**Figure 4:**
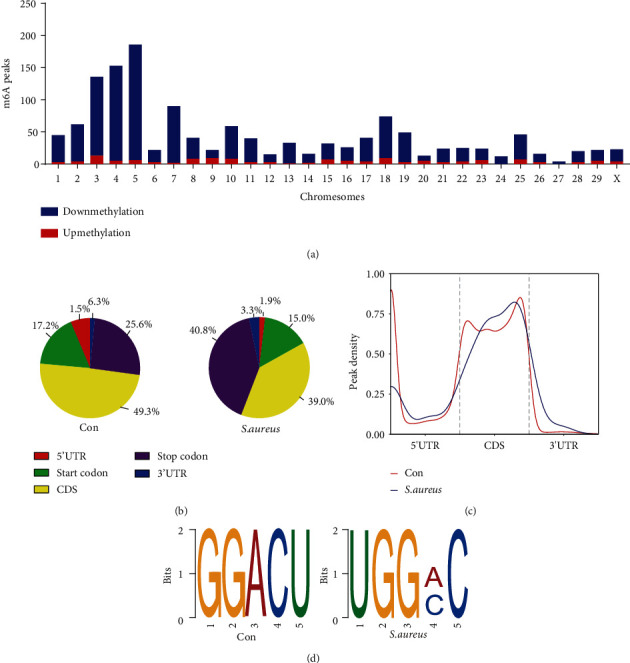
m^6^A modification map of *S. aureus*-induced mastitis. (a) The distribution of the differential m^6^A-modified genes on bovine chromosomes. (b) Gene position distribution of the differential m^6^A modification sites in the control and *S. aureus* groups. (c) Peak density distribution of the differential m^6^A modification sites in the control and *S. aureus* groups. (d) Motif sequence analysis of m^6^A-modified genes in the control and *S. aureus* groups.

Further analysis revealed the differential methylation sites on all chromosomes, especially chromosomes 3, 4, and 5 (Figure 4(a)). To obtain the preferred location distribution of the m^6^A methylation peaks in the genes, this study performed a statistical analysis of the transcriptome. We found that the m^6^A peak was mainly enriched in the coding sequence, stop codon, and 3′-untranslated regions in the two groups (Figures 4(b) and 4(c)). The most enriched motif sequence of the m^6^A peaks was GGACU in the control group and UGGAC in the *S. aureus* group (Figure 4(d)). These data are similar to those obtained in previous studies, further enhancing the reliability and authenticity of the available data [33].

### 3.4. Differentially m^6^A Methylated RNAs Were Involved in Mastitis-Related Processes

**Figure 5:**
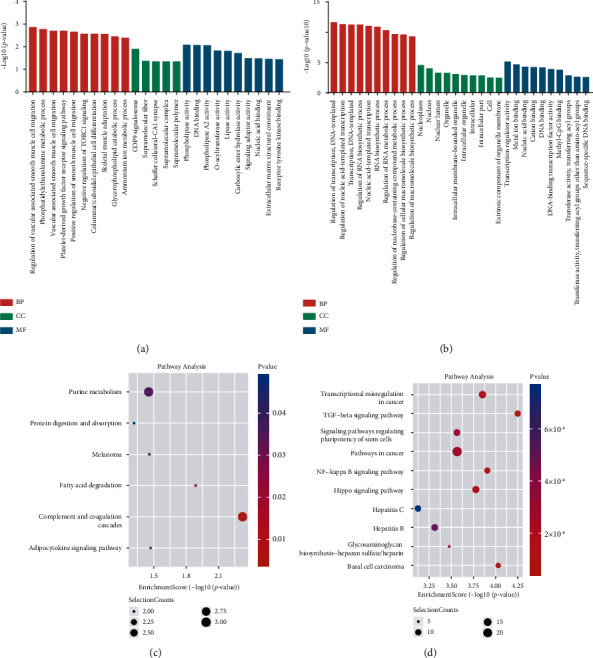
Biological functional analysis of differential m^6^A-modified genes. GO analysis of hypermethylated (a) and hypomethylated genes (b) in the *S. aureus* group. KEGG analysis of hypermethylated genes (c) and hypomethylated genes (d) in the *S. aureus* group.

To further explore the biological function of m^6^A methylation in bovine mammary epithelial cells stimulated by *S. aureus*, gene with different m^6^A peaks was analyzed by GO and KEGG analyzed. The GO analysis showed that the m^6^A hypermethylated genes in the *S. aureus* group were more closely associated with the regulation of vascular-associated smooth muscle cell migration and phosphatidylethanolamine metabolic process (biological process, [BP]), COP9 signalosome (cellular component, [CC]), and phospholipase activity (molecular function, [MF]) (Figure 5(a)). The hypomethylated genes were significantly involved in transcription regulation, DNA-templated synthesis (BP), nucleoplasm (CC), and transcription regulator activity (MF, Figure 5(b)).

Remarkably, according to the KEGG analysis, we identified that the m^6^A hypermethylation genes were significantly associated with fatty acid degradation and adipocytokine signaling pathway, amongst others (Figure 5(c)), whereas the hypomethylated genes were mainly enriched in the TGF-*β*, NF-*κ*B, and Hippo signaling pathways (Figure 5(d)), which are associated with the progression of mastitis.

### 3.5. Conjoint Analysis of Differential m^6^A Modification and mRNA

**Figure 6:**
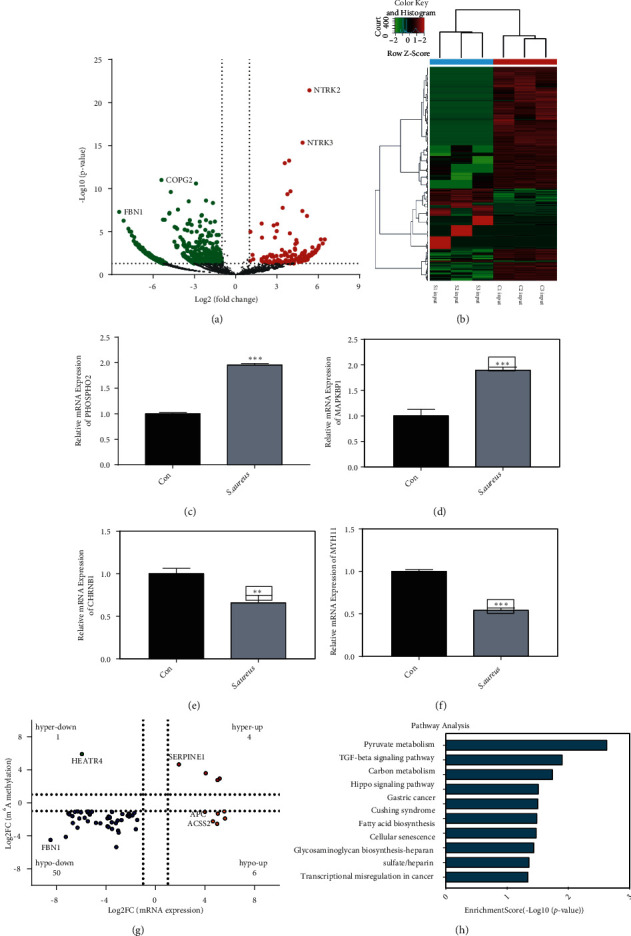
Joint analysis of differential mRNA expression and m^6^A modification. (a) Volcano map showing the differential mRNA expressions between the control and *S. aureus* groups (*p* < 0.05, fold change > 2). (b) Heat map demonstrating the differential mRNA expressions between the three control input samples and three *S. aureus* input samples. (c)–(f) mRNA expressions of PHOSPHO2, MAPKBP1, CHRNB1, and MYH11 detected by RT-qPCR. (g) Quadrant diagram showing the genes with differential m^6^A modification (*p* < 0.00001, fold change > 2) and differential mRNA expression (*p* < 0.05, fold change > 2). (h) KEGG analysis of the biological functions of 62 genes with differential m^6^A modification and mRNA expression.

RNA-seq was used to detect the mRNA expressions in the control and *S. aureus* groups (Figures 6(a) and 6(b)). Compared with the control group, 848 differentially expressed genes (DEGs) were found in *S. aureus* groups (fold change > 2 and *p* < 0.05, Table S2); among those genes, there are 249 upregulated genes and 599 downregulated genes.  Table 4 shows the top 20 DEGs in the control and *S. aureus* groups. Meanwhile, we verified that the mRNA expression of the genes PHOAPHO2 and MAPKBP1 was upregulated, and that of CHRNB1 and MYH11 was downregulated by RT-qPCR (Figures 6(c)–6(f)).

By crossanalysis of the MeRIP-seq and RNA-seq data, we discovered that in 135 hypermethylated sites (fold change > 2 and *p* < 0.05), four genes were upregulated (called “hyper-up”), and one gene was downregulated (called “hyper-down,” fold change > 2 and *p* < 0.05). In the 871 hypomethylated sites (fold change > 2 and *p* < 0.05), six genes were upregulated (called “hypo-up”), and 50 genes were downregulated (called “hypo-down,” fold change > 2 and *p* < 0.05, Figure 6(g)). We presented 15 differentially methylated and expressed genes (Table 5). The KEGG analysis of the 62 genes revealed that these genes were mainly enriched in pyruvate metabolism, the TGF-*β*, and Hippo signaling pathway (Figure 6(h)).

## 4. Discussion

In mastitis in dairy cows, pathogenic microorganisms usually induce breast inflammation. *Staphylococcus aureus* is one of the most common pathogens that often causes subclinical mastitis [34]. Previous studies have shown that bovine mammary epithelial cells are the first line of defense against the invasion of mammary glands by microorganisms such as *S. aureus* [1, 35], which leads to the release of various chemokines and cytokines [36]. Although there is currently much research into mastitis, the molecular mechanism of mastitis caused by *S. aureus* is still unclear. There are many challenges in improving mastitis diagnosis, treatment, and prevention. m^6^A methylation modification can affect RNA splicing, transcription, and translation [37–39] and thereby participating in the initiation and progression of many diseases such as cancer and cardiovascular diseases [26, 40, 41]. However, only a few studies have reported m^6^A methylation modification in mastitis. To our knowledge, this is the first study to report the m^6^A map of *S. aureus*-induced mastitis, which provides a clue for further study of m^6^A modification in mastitis.

In bovine, studies have shown that *S. aureus* induces an inflammatory response and finally leads to the secretion of cell factors, such as TNF-*α*, IL-6, and IL-1 [42, 43]. Oxidative stress is a state of imbalance between oxidation and antioxidation, that increases the production of ROS [44]. In mastitis, the release of ROS is key to the inflammatory response [45]. On the one hand, ROS plays an important role in inflammation, apoptosis, and cell growth [46, 47]. On the other hand, ROS can cause oxidation of proteins and DNA, inducing damage to nearby tissues. In this study, we used inactivated *S. aureus* to stimulate MAC-T cells, and we found that the expression levels of the inflammatory factors and ROS increased significantly (Figure 1), consistent with the results of other related studies [47, 48].

m^6^A modification is considered a reversible dynamic modification. In addition, the methylase system determined its biological function [13]. Studies have shown that most diseases are accompanied by changes in methylases [21, 23]. Wu et al. reported the mRNA expression levels of m^6^A related enzymes in bovine mammary epithelial cells treated with aflatoxins B1 and M1 [49]. In this study, the mRNA levels of methyltransferase (METLL3, METLL14, and WTAP) and a demethylase (ALKBH5) were upregulated (Figure 2), which suggests that m^6^A modification may be related to the inflammatory response and oxidative stress induced by *S. aureus* in the MAC-T cells.

With analysis the MeRIP-seq results, we speculated that *S. aureus* may have induced the m^6^A modification of some RNA molecules in MAC-T cells. Therefore, the m^6^A modification map of *S. aureus*-induced MAC-T cells was described using the MeRIP-seq technique. We found 1,006 differential methylation sites in 844 genes, some of which are closely related to the occurrence and development of mastitis. PDGFRA (Figure 3(b)), a hypermethylated molecule, reportedly induced constitutive phosphorylation of Akt, ERK1/2, and STAT3 [50]. Among the hypomethylated molecules, TNF (Figure 3(b)), a cytokine, and NF*κ*B1 and NF*κ*B2 (Table S1), which are important transcription factors, are essential for inflammation and innate immunity [51, 52]. TGF-*β*2 (Table S1) is a member of the TGF factor superfamily that plays an important role in regulating the initiation, maintenance, and resolution of immune responses and epithelial-mesenchymal transition [53, 54]. TRAF1 (Figure 3(b)) plays an important role in mediating cell survival, differentiation, proliferation, and death [54], and inhibition of TRAF1 can effectively inhibit inflammation, oxidative stress, and apoptosis [55]. We confirmed the m^6^A hypermethylation of PDGFRA and hypomethylation of TNF and TRAF1 through MeRIP-qPCR (Figure 3(c)). However, whether these molecules function through m^6^A modification requires further verification.

Abnormal lipid metabolism and oxidative stress are important factors that lead to the development of inflammatory diseases. Similar metabolic abnormalities have been reported in the early stages of mastitis [56, 57]. Researchers believed that lipids and their metabolites could be used as predictive diagnostic markers, preventive tools, and early treatment interventions for mastitis [57, 58]. In this study, GO analysis of differential m^6^A methylated genes revealed that the BP functions were mainly enriched in the phosphatidylethanolamine metabolic process, glycerophospholipid catabolic process (Figure 5(a)), and so on. The KEGG analysis revealed that the differential m^6^A methylated genes were mainly involved in the fatty acid degradation signaling pathway (Figure 5(c)). Thus, we speculated that m^6^A modification may affect the occurrence of *S. aureus*-induced mastitis through lipid metabolism and oxidative stress. In addition, previous studies have confirmed that bovine mammary epithelial cells transmit inflammatory signals mainly through the TGF, NF-*κ*B, and TNF signaling pathway and other signaling pathways [59, 60]. The KEGG pathway analysis revealed that the differential m^6^A methylated genes were also enriched in the TGF and NF-*κ*B signaling pathways (Figure 5(d)) in the current study. This suggests that m^6^A modification may be associated with mastitis. m^6^A modification is well known that affects mRNA splicing, translation, and stability [37–39]. In the GO analysis, we also found that the differential m^6^A modification was mainly related to biological progress items such as RNA biosynthetic process and transcription. Meanwhile, we conducted a joint analysis of differential m^6^A molecules and mRNA. We found 62 genes whose m^6^A modification and mRNA expression levels had changed significantly (*p* < 0.05, Figure 6(c)), and the changes in mRNA expression levels might have been caused by the change in the m^6^A modification. Biological analysis of these 62 genes revealed that they were related to pyruvate metabolism, fatty acid biosynthesis, TGF-beta signaling pathway, and so on (Figure 6(d)).

The heat-inactivated *S. aureus* mastitis model retained the main infectious components while avoiding bacterial overgrowth and excessive cell death. There are many ways to inactivate *S. aureus*, such as ultraviolet irradiation (UV) and chemical treatment. However, it has been suggested that gram-positive bacteria are more resistant to ultraviolet light than gram-negative bacteria [61, 62]. The inactivation of *S. aureus* using chemical treatment, such as formaldehyde, is not completely effective [63]. In addition, residual chemical agents may have some effect on mammalian cells during subsequent infection experiments. The heat inactivation method can not only effectively inactivate *S. aureus* but is also simple and easy to perform. Therefore, the establishment of a mastitis model with heat-inactivated *S. aureus* has been recognized and applied by many researchers [30, 64, 65].

**Figure 7:**
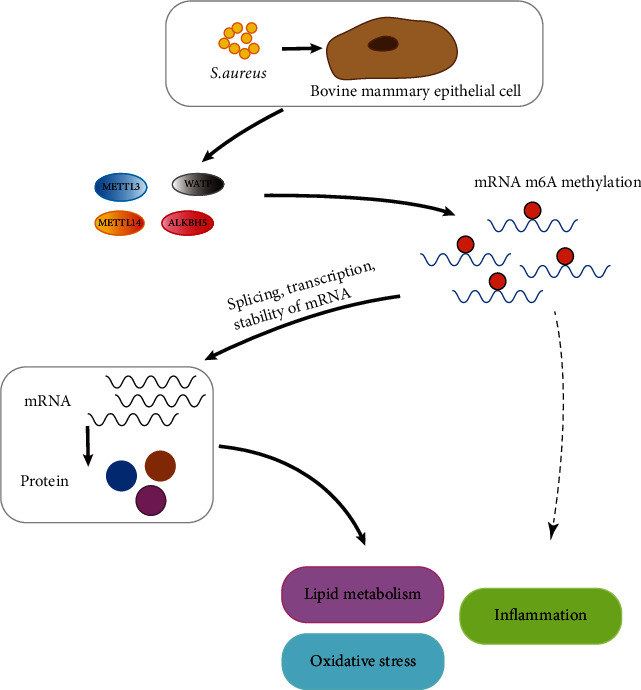
Schematic diagram of the potential mechanism of m^6^A modification in MAC-T cells treated by *S. aureus*.

Through the above analysis, we speculate that, in MAC-T cells treated with *S. aureus*, m^6^A modification will affect the transcription and translation of mRNA, thus affecting the physiological and pathological processes of inflammation, oxidative stress, and lipid metabolism (Figure 7). However, the mechanism by which m^6^A regulates mastitis in bovine is still unclear. This study thus provides a clue to the mechanism of m^6^A modification in *S. aureus*-induced mastitis and should be explored further in future studies.

## 5. Conclusions

The results of this study clearly show the changes in the m^6^A modification spectrum in *S. aureus*-induced mastitis. We found that the different m^6^A-modified molecules were involved in lipid metabolism, oxidative stress, inflammatory reactions, and other mastitis-related biological processes. This study broadens the research direction for dairy cow mastitis and lays the foundation for further research that the function of m^6^A modification in mastitis.

## Figures and Tables

**Table 1:** Primer sequence of mRNA in RT-qPCR.

Gene name	Forward primer	Reverse primer
*β*-Actin	AGATCAAGATCATCGCGCCC	TAACGCAGCTAACAGTCCGC
IL-1*β*	TTCCATATTCCTCTTGGGGTAGA	AAATGAACCGAGAAGTGGTGTT
IL-6	AGCAGGTCAGTGTTTGTGG	CTGGGTTCAATCAGGCGAT
TNF-*α*	TCTTCTCAAGCCTCAAGTAACAAGC	CCATGAGGGCATTGGCATAC
METTL3	GGAACACTGCTTGGTTGGTG	GGTTGCACATTGTGTGGTCG
METTL14	TTGGAGCAAGGGTTCATCCG	CACTTTCAGCTCCCAACTGC
FTO	CTCCGTCTGGAGAGGATTCA	TGCTCCTTGGTTGCTAGTCG
WTAP	CTCCGTCTGGAGAGGATTCA	CTGCGTGCAGATTCTTGCTG
ALKBH5	CCCATCCACATCTTCGAGCG	AGCAGCGTATCCACTGAGCAC

**Table 2:** Primer sequence of m^6^A modified part in mRNA in RT-qPCR.

Gene name	Forward primer	Reverse primer
TNF-*α*	AATTATGGGCTCAGGGCTGG	TCCTTGATGGTGGTTGGTGG
PDGFRA	GACCAGCAGGTTCTAGTCCTAAT	GCAGGAGGCCAAAAAGGAAAC
TRAF1	ATGAAGGCGGAAGGTCCAGA	CAGAGTCCACCTCCACGTTC
TLR4	CCGGCTGGTTTTGGGAGAAT	ATGGTCAGGTTGCACAGTCC

**Table 3:** The top 20 differential m^6^A-modified peaks between *S. aureus* and con based on *p* value.

Gene name	Peak region	Peak start	Peak end	Chromosome	Log10 (*p* value)	Log2 (fold change)	Hyper/hypo
KDM3A	CDS	48394594	48394800	NC_037338.1	-7.35	1.27	Hyper
C18H19orf81	StopC	56747061	56748820	NC_037345.1	-6.59	1.09	Hyper
LZTS1	CDS	67371075	67371555	NC_037335.1	-6.49	1.21	Hyper
USP26	CDS	16901441	16901740	NC_037357.1	-6.12	3.89	Hyper
CDH4	CDS	55180735	55180969	NC_037340.1	-6.07	4.30	Hyper
CRCT1	StopC	18184161	18184773	NC_037330.1	-5.98	5.74	Hyper
KLHL6	CDS	83597549	83597780	NC_037328.1	-5.86	5.90	Hyper
SPECC1	5′UTR	33638477	33638721	NC_037346.1	-5.86	1.03	Hyper
LOC781261	5′UTR	74730061	74730340	NC_037335.1	-5.69	5.94	Hyper
GAB2	3′UTR	17794541	17795150	NC_037356.1	-5.67	1.52	Hyper
LOC101903326	StopC	1089721	1090900	NC_037341.1	-8.60	8.19	Hypo
LOC616254	StopC	48235186	48235992	NC_037346.1	-8.43	8.73	Hypo
LOC100848799	CDS	52319261	52319860	NC_037334.1	-8.06	4.30	Hypo
FAM198B	StartC	40796022	40797166	NC_037344.1	-8.01	8.43	Hypo
TACR2	StopC	25769941	25770440	NC_037355.1	-7.94	4.06	Hypo
GPR132	StopC	69438921	69439920	NC_037348.1	-7.90	1.95	Hypo
FRMPD1	CDS	61863921	61865028	NC_037335.1	-7.88	3.09	Hypo
ANGPT4	StopC	60234003	60234780	NC_037340.1	-7.88	8.61	Hypo
OR9Q2	CDS	81371461	81372540	NC_037342.1	-7.87	5.35	Hypo
SAA3	CDS	26414395	26414676	NC_037356.1	-7.87	4.68	Hypo

**Table 4:** The top 20 differential mRNA expression in *S. aureus* vs. con.

gene_id	LogFC	Log10 (*p* value)	Regulation
ADGRE3	6.49	-4.09	Up
STARD7	6.33	-3.63	Up
TRIP11	6.20	-4.10	Up
NTRK1	6.06	-3.36	Up
KRT80	6.02	-3.19	Up
LOC527796	6.01	-3.05	Up
RAB4B	5.88	-2.80	Up
MAPKBP1	5.85	-2.77	Up
PHOSPHO2	5.81	-2.84	Up
LOC100849008	5.81	-2.72	Up
FBN1	-8.47	-7.29	Down
MYH11	-8.15	-6.27	Down
ACP2	-7.80	-5.34	Down
PRR29	-7.71	-5.01	Down
LOC112444598	-7.59	-4.99	Down
CHRNB1	-7.55	-4.56	Down
PSIP1	-7.41	-3.92	Down
DXO	-7.37	-4.36	Down
DSG1	-7.29	-3.61	Down
GLS	-7.25	-3.58	Down

**Table 5:** 15 transcripts of differential m^6^A modification and mRNA expression in *S. aureus* vs. con.

Gene name	Change	Chromosome	m^6^A modification change						mRNA expression change	
			Peak start	Peak end	Peak_length	Peak region	Fold change	Log10 (*p* value)	LogFC	Log10 (*p* value)
ALK	Hyperup	NC_037338.1	70660375	70661760	1385	CDS	7.70	-5.10	5.19	-6.82
ERO1A	Hyper-u	NC_037337.1	11498514	11498840	326	5′UTR	5.13	-5.13	5.02	-2.55
RYBP	Hyperup	NC_037349.1	29277201	29277420	219	3′UTR	5.21	-5.21	4.06	-1.41
SERPINE1	Hyperup	NC_037352.1	35605621	35605860	239	5′UTR	5.22	-5.22	1.90	-1.81
HEATR4	Hyperdown	NC_037337.1	84967915	84968140	225	5′UTR	5.15	-5.15	-5.94	-1.89
FBN1	Hypodown	NC_037337.1	61917881	61918380	499	CDS	-22.53	-7.75	-8.47	-7.29
CD70	Hypodown	NC_037334.1	17908292	17908687	395	CDS	-17.43	-5.40	-7.24	-3.51
LHFPL4	Hypodown	NC_037349.1	17088303	17088792	489	CDS	-2.46	-5.03	-7.03	-3.19
KMT2E	Hypodown	NC_037331.1	46268266	46268531	265	CDS	-2.37	-6.93	-6.96	-3.26
PRPF38A	Hypodown	NC_037330.1	93935321	93935880	559	3′UTR	-2.63	-6.14	-6.95	-3.02
EXT1	Hypoup	NC_037341.1	46477327	46477877	550	CDS	-2.05	-6.07	5.56	-2.60
RPGR	Hypoup	NC_037357.1	104982661	104983554	893	CDS	-2.47	7.23	5.04	-1.69
ARSI	Hypoup	NC_037334.1	61646441	61648420	1979	3′UTR	-5.82	-5.60	4.99	-1.67
ACSS2	Hypoup	NC_037340.1	64232901	64233260	359	3′UTR	-4.76	-5.39	4.64	-1.50
APC	Hypoup	NC_037334.1	43874981	43877340	2359	CDS	-4.16	-5.01	3.99	-9.68

## Data Availability

The data obtained in this study are available from the corresponding author upon request.

## Supplementary Materials

Supplementary Materials Table S1: differentially methylated RNA sites for *S. aureus* and con. Table S2: differential mRNA expression for *S. aureus* and Con.
