# Supplementary material for: N6-Methyladenosine Modification Profile in Bovine Mammary Epithelial Cells Treated with Heat-Inactivated Staphylococcus aureus
Source: Oxid Med Cell Longev. 2022 Feb 23;2022:1704172. doi: 10.1155/2022/1704172 (PMC8890870; doi:10.1155/2022/1704172)
Supplement: Supplementary Materials — Table S1: differentially methylated RNA sites for S. aureus and con. Table S2: differential mRNA expression for S. aureus and Con. [file 1704172.f1.zip › Table S1.pdf]

Table S1. Differentially methylated RNA sites for: *S. aureus* _vs_Con<br>Fold change cut-off: 2<br>P-value cut-off: 0.00001							
**GeneName**	**txStart**	**txEnd**	**Chrom**	**Peak_ length**	**log2(FoldChange)**	**lg(P-value)**	**Regulation**
POLA2	43504012	43504240	29	228	2.544720654	-5.04412566	hyper
RYBP	29277201	29277420	22	219	3.584962501	-5.213292467	hyper
FOXD1	8183421	8183800	20	379	5.935459748	-5.034591042	hyper
KDM3A	48394594	48394800	11	206	1.270224874	-7.348507483	hyper
TCTE1	17783681	17783900	23	219	5.754887502	-5.473179528	hyper
PNPLA8	49588601	49588820	4	219	3.86507042	-5.067130576	hyper
LOC788175	12133081	12133480	3	399	5.744161096	-5.216175115	hyper
ALK	70660375	70661760	11	1385	2.94428644	-5.096748648	hyper
LZTS1	67371075	67371555	8	480	1.212695224	-6.494254234	hyper
ERO1A	11498514	11498840	10	326	2.752990467	-5.134181229	hyper
ADM	42378001	42378320	15	319	5.935459748	-5.028754579	hyper
LOC785244	36701	36920	NW_020191779.1	219	2.830941742	-5.286446323	hyper
LIX1L	21440946	21441280	3	334	2.125745553	-5.009581431	hyper
GVQW3	55823141	55823460	15	319	5.916476644	-5.147675438	hyper
COPS7B	119526921	119527280	2	359	2.438039325	-5.045388592	hyper
IL31RA	23279841	23280180	20	339	5.935459748	-5.028754579	hyper
DEGS2	65093861	65094254	21	393	2.569132272	-5.034065933	hyper
LOC112443853	6727101	6727460	23	359	2.919304766	-5.501066292	hyper
KLHL6	83597549	83597780	1	231	5.897240426	-5.859800787	hyper
LOC112447503	9744081	9744336	7	255	5.935459748	-5.028754579	hyper
SPECC1	33638477	33638721	19	244	1.03332342	-5.857030656	hyper
LOC781152	37821	38119	NW_020191248.1	298	3.136372442	-5.100143325	hyper
LURAP1	99869481	99869780	3	299	5.532940288	-5.233182794	hyper
RBAK	38548941	38549280	25	339	5.916476644	-5.147675438	hyper
CHRM1	41232881	41233187	29	306	5.935459748	-5.034591042	hyper
EFNB2	82288801	82289093	12	292	2.59285041	-5.094507393	hyper
LOC513659	53888061	53888440	3	379	5.744161096	-5.216175115	hyper
CPT1A	46169281	46169520	29	239	4.778973121	-5.117135022	hyper
CFAP69	74502810	74503100	4	290	4.413157766	-5.23418826	hyper
LOC786870	47817404	47817635	15	231	3.070389328	-5.001968767	hyper
FOSB	53076964	53077285	18	321	5.095829659	-5.377628932	hyper
ALDH1B1	62434981	62435340	8	359	5.744161096	-5.501700188	hyper
CASTOR1	69305133	69305374	17	241	2.52501996	-5.152415599	hyper
LOC104968438	23103181	23103386	8	205	5.935459748	-5.028754579	hyper
RHEBL1	30752381	30752760	5	379	5.741466986	-5.504099672	hyper
LOC112445067	37915341	37915640	30	299	4.6794801	-5.588430167	hyper
LOC101903205	29789521	29789900	8	379	5.916476644	-5.147675438	hyper
CAMKK2	53888921	53889149	17	228	2.494232725	-5.202553729	hyper
TMEM138	39868421	39868800	29	379	3.483517237	-5.181509011	hyper
DHX34	54342722	54343000	18	278	4.653442239	-5.106502485	hyper
F2RL2	7854380	7854604	10	224	2.101235295	-5.007865992	hyper
LOC785723	27768801	27769020	10	219	5.741466986	-5.504099672	hyper
BASP1	55908759	55909060	20	301	2.066342495	-5.13493048	hyper
RBFOX3	52892528	52893000	19	472	2.573674429	-5.27609034	hyper
SERPINE1	35605621	35605860	25	239	4.653442239	-5.215978191	hyper
RAB3IP	43712841	43713120	5	279	3.562242424	-5.0106999	hyper
LOC112443853	6752921	6753260	23	339	5.916476644	-5.147675438	hyper
PDGFRA	69770501	69770720	6	219	4.128104826	-5.128618679	hyper
LOC104968434	92832861	92833200	4	339	3.672425342	-5.173430332	hyper
MUC3A	35725301	35725600	25	299	5.935459748	-5.034591042	hyper

ZNF34	308121	308480	14	359	3.03433204	-5.193128073	hyper
LOC789121	105600877	105601200	4	323	5.935459748	-5.028754579	hyper
IGFN1	79731341	79731560	16	219	5.5360529	-5.236841646	hyper
RAB2B	25693006	25693300	10	294	1.744236121	-5.008348045	hyper
BEAN1	34232281	34232760	18	479	2.18663346	-5.069465297	hyper
STARD9	37997641	37998080	10	439	3.39714734	-5.047630368	hyper
ZBED2	56343341	56343580	1	239	3.672425342	-5.164714721	hyper
FGF14	78131961	78132180	12	219	5.935459748	-5.028754579	hyper
LOC112441491	64657057	64657280	18	223	6.106432078	-5.232980408	hyper
IL-15L	49130621	49130860	18	239	5.532940288	-5.233182794	hyper
ZEB2	52263120	52263380	2	260	5.741466986	-5.218511649	hyper
CSRNP1	12488421	12488900	22	479	2.103537604	-5.060196798	hyper
MGC134232	124050801	124051180	30	379	5.722466024	-5.515091496	hyper
C18H19orf81	56747061	56748820	18	1759	1.093787147	-6.590297725	hyper
PIGM	9647521	9647791	3	270	1.259790831	-5.075133557	hyper
FAM177B	26239581	26239925	16	344	2.72357943	-5.045165217	hyper
LOC112446614	59005076	59005300	5	224	5.752213368	-5.47416502	hyper
ISCA2	85785621	85785880	10	259	4.018859027	-5.290767635	hyper
LOC112443209	716321	716560	21	239	3.925999419	-5.13448304	hyper
PALB2	21292261	21292540	25	279	5.5360529	-5.236841646	hyper
SH2B2	34700155	34700425	25	270	1.344495852	-5.006395764	hyper
CSRNP1	12489961	12490240	22	279	5.495055528	-5.169805904	hyper
PDE6C	14912461	14912820	26	359	5.754887502	-5.473179528	hyper
PANK1	11302181	11302737	26	556	1.217190283	-5.063678996	hyper
CFLAR	89725961	89726258	2	297	2.883071976	-5.164338945	hyper
LOC786254	106067361	106067740	4	379	3.584962501	-5.014604719	hyper
INTS1	41444829	41445084	25	255	1.068479738	-5.429283368	hyper
TTC23	7417841	7418180	21	339	3.565979397	-5.197093036	hyper
ELAVL4	96071528	96071820	3	292	5.916476644	-5.147675438	hyper
LOC788535	59529681	59529920	5	239	3.594361199	-5.165101554	hyper
MAP1B	9405421	9405642	20	221	3.461133914	-5.307482581	hyper
PSORS1C2	28007101	28007618	23	517	2.108040629	-5.096674371	hyper
KHDRBS3	6543508	6543760	14	252	3.702798991	-5.174177158	hyper
DUSP27	1607521	1607860	3	339	2.456189606	-5.191290024	hyper
ANGPTL6	14658021	14658322	7	301	5.935459748	-5.028754579	hyper
NOL9	46834190	46834400	16	210	2.819427754	-5.144205124	hyper
LOC100847679	14961	15240	NW_020190791.1	279	5.935459748	-5.028754579	hyper
IFI16	10746901	10747260	3	359	3.633217669	-5.191608248	hyper
COL11A1	40529801	40530080	3	279	5.935459748	-5.028754579	hyper
LOC532479	78251901	78252260	15	359	5.5360529	-5.236841646	hyper
CLPTM1L	71117510	71117880	20	370	2.341766842	-5.386201766	hyper
HAO2	23733641	23733960	3	319	2.936005146	-5.537707785	hyper
IGFBP5	104679441	104679740	2	299	4.102238194	-5.25895877	hyper
LOC506121	77916561	77916898	15	337	3.672425342	-5.164714721	hyper
F5	37185681	37186060	16	379	5.935459748	-5.028754579	hyper
LOC112442402	47705461	47705900	18	439	4.109121722	-5.205686668	hyper
FHAD1	52086473	52086760	16	287	5.935459748	-5.028754579	hyper
MCOLN2	59173601	59173980	3	379	5.741466986	-5.218511649	hyper
CRCT1	18184161	18184773	3	612	5.741386727	-5.977107255	hyper
CDH4	55180735	55180969	13	234	4.295575576	-6.069287097	hyper
USP26	16901441	16901740	30	299	3.892923108	-6.121177228	hyper
NEFH	68691661	68692040	17	379	3.171666335	-5.197966222	hyper
NQO2	50632041	50632639	23	598	1.001658842	-5.374273218	hyper
NAV3	7681281	7681780	5	499	1.378511623	-5.4720769	hyper
LOC101902555	516281	516660	NW_020190994.1	379	4.028951374	-5.215387871	hyper
CDC6	40535001	40535309	19	308	3.192400034	-5.123191925	hyper

PHYHIPL	14882021	14882440	28	419	4.128104826	-5.135067485	hyper
IL1RL2	7057361	7057700	11	339	3.502500341	-5.139343023	hyper
PDCD1	120838721	120839000	3	279	2.788685711	-5.142370799	hyper
LOC781261	74730061	74730340	8	279	5.942514505	-5.692696738	hyper
ADPRHL1	86660787	86661020	12	233	5.935459748	-5.028754579	hyper
GAB2	17794541	17795150	29	609	1.522340458	-5.671550211	hyper
ENTPD7	20656021	20656320	26	299	2.077494521	-5.202627903	hyper
LOC100299268	51613621	51613940	18	319	5.942514505	-5.416192589	hyper
TMEM110	48000341	48000556	22	215	2.802516365	-5.204364925	hyper
LIPH	81681395	81681680	1	285	4.263034406	-5.421792954	hyper
FAM46A	21497816	21498260	9	444	1.61693489	-5.28323943	hyper
SLC35F3	7136854	7137074	28	220	5.916476644	-5.147675438	hyper
MOB3B	16926751	16926954	8	203	1.324091867	-5.118184718	hyper
ZNF394	36908941	36909440	25	499	2.6604527	-5.061602111	hyper
RFX3	40970201	40970580	8	379	3.469969978	-5.08660388	hyper
LOC788263	47313301	47313661	15	360	2.870670408	-5.514970469	hyper
HNRNPF	13809861	13810260	28	399	1.925809102	-5.031569184	hyper
HEATR4	84967915	84968140	10	225	5.916476644	-5.147675438	hyper
LOC107131258	26328701	26329060	23	359	2.71596199	-5.220441675	hyper
LOC100337392	45914561	45914940	15	379	5.916476644	-5.147675438	hyper
FNIP2	40197537	40197780	17	243	3.05087233	-5.267041868	hyper
PPM1K	36449276	36449740	6	464	3.969083923	-5.040595636	hyper
PLA2G15	35624572	35624811	18	239	4.109121722	-5.228736878	hyper
NR4A3	64872306	64872841	8	535	3.279116515	-5.19345951	hyper
POLR3H	112629153	112629520	5	367	1.795887094	-5.069656414	hyper
CMYA5	10726601	10727040	10	439	4.128104826	-5.135067485	hyper
ATP1B4	4934692	4934900	30	208	5.722466024	-5.513673098	hyper
ENAM	86003461	86003840	6	379	4.191426071	-5.15121647	hyper
PRDM1	43864287	43864536	9	249	3.12657754	-5.535305193	hyper
GOLGA3	44466921	44467240	17	319	-1.428167544	-7.052437896	hypo
CHD8	25769821	25770783	10	962	-1.026150492	-5.059181906	hypo
PCDHGA2	52455181	52456287	7	1106	-2.099600958	-5.367335648	hypo
LOC100848991	58759742	58760200	18	458	-1.213171555	-5.160352094	hypo
CGNL1	52767161	52767840	10	679	-3.339736552	-5.05280164	hypo
DCBLD1	33250961	33251806	9	845	-1.380468828	-5.034649657	hypo
WNT5A	45553086	45553379	22	293	-1.354391281	-6.646034128	hypo
SH3RF2	57275082	57275414	7	332	-1.332755165	-5.681022392	hypo
RHOBTB2	70315581	70315890	8	309	-2.250643824	-7.117718765	hypo
BTBD3	5466181	5466741	13	560	-1.260218408	-6.386586942	hypo
PCDHGA2	52493284	52493980	7	696	-1.318161712	-5.92819293	hypo
LOC101904667	17434328	17435346	8	1018	-2.203394075	-6.798892233	hypo
LYL1	12478141	12479660	7	1519	-1.887943682	-7.1203412	hypo
LRFN3	46636505	46637139	18	634	-2.777961137	-6.719192676	hypo
ZNF362	120410441	120411849	2	1408	-3.871843649	-7.08910195	hypo
ZNF629	26899701	26901020	25	1319	-1.858995552	-5.687979885	hypo
EPC1	33214573	33214926	13	353	-2.514144336	-5.671512031	hypo
SAV1	43552321	43552580	10	259	-1.100038537	-5.699531015	hypo
FAM212A	50430254	50431096	22	842	-1.302309175	-6.108207632	hypo
POP7	35885040	35885723	25	683	-1.164622012	-6.684983613	hypo
GABPB1	59966045	59966460	10	415	-2.091593592	-6.020930243	hypo
GTF3C6	39366981	39367381	9	400	-4.15204082	-7.697804155	hypo
ZNF48	26577034	26579060	25	2026	-1.833480807	-6.214084157	hypo
NISCH	48370593	48371826	22	1233	-1.422427077	-5.180905404	hypo
BIVM	79039769	79040367	12	598	-1.529576443	-5.057020321	hypo
PCLO	37653075	37654120	4	1045	-1.825993558	-5.070945039	hypo
H2AFY	46804741	46805760	7	1019	-1.645415018	-5.374931777	hypo
RNF14	53044387	53044915	7	528	-1.134453287	-6.4678026	hypo
LOC787554	57752401	57753180	18	779	-1.667958145	-6.806414711	hypo

C10H14orf93	21857877	21858614	10	737	-2.143512326	-5.408202389	hypo
PC	44866414	44866636	29	222	-1.800769025	-6.270578832	hypo
GTF2IRD2	32749181	32750280	25	1099	-1.515474106	-5.087698179	hypo
ATP8B3	44124706	44125254	7	548	-2.682410395	-5.661512382	hypo
CYP2U1	17263221	17264295	6	1074	-2.307828345	-5.765273086	hypo
SMIM33	50735821	50736420	7	599	-3.605324662	-7.055516653	hypo
MZF1	65786621	65788180	18	1559	-1.360734428	-5.819533944	hypo
PCYOX1L	60766578	60767400	7	822	-2.25228114	-5.448005724	hypo
NGRN	21640616	21641460	21	844	-1.22255051	-6.667680594	hypo
SWT1	66185812	66186460	16	648	-1.819588274	-5.54308801	hypo
LOC616254	48249684	48250188	19	504	-4.054397088	-7.438602677	hypo
LINS1	6056021	6056280	21	259	-3.038860256	-5.823987114	hypo
PCDHGC3	52565850	52566460	7	610	-3.029226139	-6.027129117	hypo
ADNP	78822001	78822520	13	519	-1.542377819	-6.034301802	hypo
ZNF672	42627633	42629560	7	1927	-1.748518503	-6.177944938	hypo
ST3GAL5	49034215	49034620	11	405	-2.069974164	-6.651852378	hypo
TMEM200A	68537650	68537860	9	210	-2.157834825	-7.298937858	hypo
NLGN1	93148881	93149587	1	706	-1.647874784	-6.516927057	hypo
USP40	113174905	113175760	3	855	-1.544089482	-6.607146469	hypo
SMTNL2	25103813	25104056	19	243	-1.475535839	-6.624599993	hypo
FBXL4	50878775	50879088	9	313	-3.060959915	-5.109666995	hypo
FSD2	23059108	23059814	21	706	-3.289577895	-7.646816498	hypo
HOXB3	37938677	37939274	19	597	-4.427506851	-7.836555557	hypo
IFIH1	34111841	34112180	2	339	-4.712646963	-5.009390924	hypo
ANKRD17	88215901	88216197	6	296	-1.320931261	-6.507368823	hypo
TIA1	68516241	68516980	11	739	-2.047433562	-6.550154044	hypo
CCDC88A	38037220	38037498	11	278	-1.167758125	-6.270020713	hypo
LOC112441465	54447	55400	NW_020192290.1	953	-1.261867379	-6.182214791	hypo
NIPBL	37165621	37166180	20	559	-1.556474086	-5.632707752	hypo
ANKRD27	43093681	43094120	18	439	-1.252581598	-6.410283318	hypo
YIPF5	55260101	55260517	7	416	-1.677333303	-5.015936657	hypo
SMAD1	12760634	12760892	17	258	-1.585302222	-5.309838636	hypo
RHOBTB2	70304749	70305774	8	1025	-1.809423092	-6.50029059	hypo
ADAMTS6	14332221	14332760	20	539	-2.360621138	-7.201696429	hypo
PCDHGA2	52449101	52450343	7	1242	-1.582862677	-7.072860133	hypo
UBR4	133588917	133589505	2	588	-1.123031472	-6.401641519	hypo
LOC404051	50772853	50774989	23	2136	-2.511708188	-5.776257746	hypo
NUDT2	75734821	75735276	8	455	-1.888169619	-5.949399496	hypo
LOC616254	48204341	48205197	19	856	-3.25836759	-5.327798546	hypo
MLH3	86296841	86297360	10	519	-3.614805746	-6.252162715	hypo
LOC100848264	112836	113700	27	864	-1.181266063	-5.302347816	hypo
IL17RD	44001021	44001886	22	865	-1.248511298	-6.970855083	hypo
FAM120B	104016321	104016960	9	639	-3.36390529	-5.154164329	hypo
ZBTB37	55249761	55251040	16	1279	-1.82853365	-6.354841724	hypo
AUTS2	29783941	29785459	25	1518	-3.040961173	-7.222697484	hypo
SCN1A	30317701	30319040	2	1339	-1.52895173	-5.166488175	hypo
QPRT	26491478	26491965	25	487	-2.733481204	-5.428904652	hypo
RIMS2	60789672	60789900	14	228	-1.953858218	-5.409897945	hypo
STARD13	28011073	28011520	12	447	-1.30901598	-5.234173487	hypo
FAM19A3	30599241	30600136	3	895	-2.9274875	-5.904511153	hypo
CHST3	28096321	28097380	28	1059	-1.073555298	-5.72350155	hypo
CREB3L2	101652058	101652275	4	217	-1.500568116	-5.424767944	hypo
ZBTB38	127531661	127532040	1	379	-2.068145254	-5.096570922	hypo
KRT15	41725881	41726453	19	572	-2.705039849	-5.207425552	hypo
CHAMP1	87094821	87095920	12	1099	-1.898177733	-6.347839693	hypo
SVIL	34658759	34659435	13	676	-1.282027582	-5.013629805	hypo
SHISAL1	114933821	114935060	5	1239	-1.164445072	-5.445480745	hypo

PCDHGA2	52479981	52482060	7	2079	-1.93301445	-5.395248203	hypo
KDM3B	49779083	49779285	7	202	-1.824888272	-5.49624498	hypo
SMARCA2	42399285	42399503	8	218	-1.302623034	-5.399332863	hypo
RNF7	127215261	127215750	1	489	-1.517909138	-5.354213983	hypo
PHF2	84988697	84989088	8	391	-2.070266039	-5.51490888	hypo
GPR68	55998981	56001240	21	2259	-1.709011709	-6.947415276	hypo
FAT2	62750681	62751100	7	419	-3.220014318	-7.328716333	hypo
WNT5A	45557541	45557780	22	239	-1.48416075	-5.263846234	hypo
FAT2	62700961	62701459	7	498	-3.457716167	-7.63531905	hypo
BMPR1B	29374521	29374880	6	359	-2.389233422	-7.414887212	hypo
AK1	98539801	98540254	11	453	-1.087554871	-7.1782611	hypo
BICDL1	62470829	62472165	17	1336	-1.144658243	-5.223882579	hypo
ZNF33B	12828181	12829180	28	999	-2.469043213	-7.199865989	hypo
APEX2	92482821	92483780	30	959	-1.441029936	-6.457768521	hypo
GOLGB1	66176846	66178740	1	1894	-1.000705741	-5.14743787	hypo
NXPE3	46164521	46165340	1	819	-2.203737755	-5.656229483	hypo
CXXC5	50885336	50885560	7	224	-2.036917859	-5.184158036	hypo
PCDHGA2	52488701	52490560	7	1859	-1.933926334	-6.821976044	hypo
RASSF6	88622881	88623178	6	297	-2.90276906	-5.789824603	hypo
ARRDC3	90841601	90842294	7	693	-3.897610329	-6.947613804	hypo
TSC22D1	14800261	14802120	12	1859	-1.080892927	-5.644349975	hypo
ADAT1	2890621	2891883	18	1262	-1.286117234	-7.016209136	hypo
ZFP41	1334251	1335220	14	969	-1.952166569	-6.600196351	hypo
CPM	44957366	44958820	5	1454	-1.872716795	-6.475772628	hypo
LPAR2	3706927	3707940	7	1013	-1.20487297	-6.176424264	hypo
TNFRSF21	20419301	20419632	23	331	-1.324094663	-5.793053135	hypo
ESPN	46916463	46916727	16	264	-3.095157233	-7.302362753	hypo
GLUL	63469241	63469740	16	499	-1.628058152	-6.446877621	hypo
DSG3	25670569	25670817	24	248	-1.417500432	-6.137678228	hypo
PML	34575881	34577657	21	1776	-1.242367827	-6.68215292	hypo
SREBF1	34648441	34649200	19	759	-1.264623145	-7.093788128	hypo
AGPAT1	27216141	27216840	23	699	-1.562437242	-6.876771566	hypo
LOC100848799	52319261	52319860	7	599	-4.301869235	-8.061056492	hypo
ZNF879	2337681	2338277	7	596	-3.046350316	-6.002250573	hypo
FAM131B	106739321	106739540	4	219	-2.654691591	-5.085817249	hypo
ZNF436	129483381	129484100	2	719	-1.730497819	-5.377719549	hypo
FZR1	20384761	20385890	7	1129	-1.121036138	-6.078906374	hypo
IFNT3	22583081	22583460	8	379	-2.441328347	-5.1111399	hypo
LOC509034	86905421	86907360	10	1939	-1.515033582	-5.386221595	hypo
ZNF398	112282461	112283480	4	1019	-1.506479094	-5.446380373	hypo
SLIT2	40182648	40183640	6	992	-2.930714353	-7.321085461	hypo
LOC112445429	13413	13800	NW_020 190482.1	387	-1.793613843	-5.392404071	hypo
KRT6A	27384761	27384980	5	219	-1.106955341	-6.296008734	hypo
LOC512672	28504701	28505222	23	521	-1.962967293	-5.114584405	hypo
CTDSP1	106412733	106413200	2	467	-2.470980251	-6.546638106	hypo
KANK1	43798814	43800140	8	1326	-1.995378442	-6.296326293	hypo
FOXN1	20001368	20001860	19	492	-2.949545285	-7.233336368	hypo
CASP4	3269041	3269300	15	259	-2.01729901	-5.185842361	hypo
DGKA	57353449	57353900	5	451	-1.42036604	-5.020905835	hypo
ABO	104177521	104178340	11	819	-3.218190476	-5.694329081	hypo
ZNF699	14193801	14194300	7	499	-2.248486546	-5.853561225	hypo
LRP4	76788761	76789809	15	1048	-1.32238544	-7.03396718	hypo
THRAP3	109553441	109554026	3	585	-1.003147861	-5.150185847	hypo
GAA	52482890	52483760	19	870	-1.997164235	-5.440597546	hypo
RHOV	36410981	36411525	10	544	-1.989277121	-5.048089186	hypo
LOC101903385	65803501	65805430	18	1929	-2.196545882	-7.430367747	hypo
ELAC1	50486701	50487340	24	639	-2.398993799	-7.386320401	hypo

GALNT10	65702401	65702920	7	519	-1.089255099	-6.133759389	hypo
ZC3H10	57199161	57200875	5	1714	-1.274650315	-5.199089467	hypo
GID8	54464732	54465541	13	809	-1.125193764	-6.233820765	hypo
TCF12	53025341	53025721	10	380	-1.535523015	-7.265106008	hypo
ZDHHC14	94301451	94302160	9	709	-2.534468069	-6.719638326	hypo
PCLO	37655141	37656820	4	1679	-1.547132284	-5.101059486	hypo
ZSCAN29	55118024	55118600	21	576	-1.715192024	-5.217767243	hypo
PARP14	67048941	67049521	1	580	-1.391805193	-5.419881715	hypo
PCLO	37656881	37657900	4	1019	-2.8547187	-7.233168787	hypo
TDRD7	62707814	62708317	8	503	-1.151500845	-5.043484899	hypo
ZNF189	91130381	91131040	8	659	-1.906056133	-5.514357038	hypo
TBCEL	31710835	31711100	15	265	-2.489948045	-5.711957559	hypo
USP32	12776618	12777043	19	425	-1.324915974	-7.093319489	hypo
FAM114A1	58099794	58100150	6	356	-1.230343674	-5.441185501	hypo
MOCS3	78872161	78873020	13	859	-1.511722013	-5.119237862	hypo
TOR3A	60450181	60450680	16	499	-2.244706829	-5.174331133	hypo
VEZF1	8784801	8785348	19	547	-1.307672563	-5.123181949	hypo
LOC616254	48203421	48204000	19	579	-3.175212833	-6.889027149	hypo
SPATS2	30456719	30457061	5	342	-1.723681371	-7.2808377	hypo
GLT8D1	48168886	48169414	22	528	-1.654499165	-5.288556541	hypo
IQCE	40709997	40711120	25	1123	-1.693065909	-6.567535124	hypo
TP53	27379912	27380167	19	255	-2.259919726	-6.930330945	hypo
ITGB8	29115099	29115480	4	381	-2.45869378	-5.379913015	hypo
MAGED2	92288221	92288463	30	242	-2.260255575	-5.862662728	hypo
HEATR6	14133061	14133687	19	626	-1.05468491	-5.796318592	hypo
GLYCTK	48590041	48590941	22	900	-2.604926431	-7.469063703	hypo
LOC782688	27769347	27770080	29	733	-1.526671989	-5.511407912	hypo
TTYH3	40748396	40749520	25	1124	-1.032097115	-5.516949627	hypo
MYO5A	57992607	57992847	10	240	-1.600131485	-5.328051866	hypo
FLYWCH1	2352981	2353440	25	459	-1.442996475	-5.46462142	hypo
PIK3IP1	70094941	70095260	17	319	-2.679316427	-5.215943314	hypo
NACAD	76545121	76546580	4	1459	-8.388017285	-7.657039282	hypo
VEZF1	8782602	8782804	19	202	-1.281582416	-6.195305404	hypo
DNAJC28	2028921	2030540	1	1619	-2.238182198	-5.660356825	hypo
CNOT2	43134290	43134660	5	370	-1.343313847	-7.451538773	hypo
KIAA1143	16500741	16501083	22	342	-1.634638128	-6.104043324	hypo
NCOA6	64072081	64073099	13	1018	-1.064396439	-6.930290042	hypo
NMT2	29682321	29683456	13	1135	-1.13650959	-6.380375197	hypo
CHD2	14043961	14044440	21	479	-1.893445619	-6.965201771	hypo
ZNF469	13703501	13709920	18	6419	-3.395047487	-5.679064909	hypo
PLEKHD1	81276041	81276880	10	839	-2.48570332	-6.996910047	hypo
PDGFC	42479081	42479520	17	439	-2.29730181	-5.807939364	hypo
MBLAC1	36331801	36333031	25	1230	-1.277567524	-7.03530189	hypo
LOC112449367	11212141	11213008	13	867	-1.272938749	-5.416279256	hypo
ZDHHC8	73013461	73014360	17	899	-1.130789444	-6.057774551	hypo
IRF1	21944841	21945380	7	539	-3.006619701	-5.375662387	hypo
SHLD2	41727641	41728780	28	1139	-2.082149041	-5.465850806	hypo
MB21D2	74608690	74609955	1	1265	-1.195620496	-7.518320714	hypo
FOXP2	53770721	53771260	4	539	-2.102199819	-5.671056831	hypo
YAF2	38537721	38538380	5	659	-2.081912483	-6.603034482	hypo
EDNRA	10597821	10598376	17	555	-2.409534409	-5.064704319	hypo
CEBPA	43728247	43729478	18	1231	-1.53906088	-6.296649272	hypo
KLHDC10	93922335	93922780	4	445	-1.140285964	-5.139756799	hypo
RXRA	105113941	105114880	11	939	-1.020873237	-5.383940747	hypo
HSPBAP1	67064461	67065078	1	617	-1.568160487	-7.239822832	hypo
IMMP1L	62219261	62220020	15	759	-1.370915269	-5.702178909	hypo
ANKRD39	2808141	2809028	11	887	-1.014836802	-7.555434796	hypo
NR2F1	93061046	93062000	7	954	-1.003901383	-6.512896399	hypo

CELSR1	116796323	116799460	5	3137	-1.201447265	-6.236037257	hypo
ZBTB38	127532121	127532380	1	259	-1.787507077	-6.982127662	hypo
LDLRAD4	43477026	43478080	24	1054	-2.421517208	-5.023994284	hypo
ZFHX2	21418646	21419860	10	1214	-1.945082558	-5.360773708	hypo
NAT1	39015901	39016322	27	421	-1.593713711	-6.035518383	hypo
ZSWIM3	74637195	74637940	13	745	-1.509518398	-5.020153809	hypo
TRIM65	55731801	55732300	19	499	-2.298501773	-5.360214491	hypo
NAV2	24802681	24804435	29	1754	-1.620341231	-5.519260829	hypo
ZNF3	36376621	36377100	25	479	-2.136329032	-6.575820667	hypo
LOC107133032	18204961	18205900	13	939	-2.791335937	-7.219949701	hypo
SH3D19	6676404	6676669	17	265	-2.192403708	-7.155579178	hypo
PCDHGA2	52485101	52486547	7	1446	-1.568049118	-5.480614465	hypo
SLC24A1	12546362	12547292	10	930	-3.130687363	-5.248095227	hypo
C25H16orf89	4070000	4070353	25	353	-2.36532635	-6.912546906	hypo
PCLO	37685641	37686857	4	1216	-2.70497183	-5.952550078	hypo
HS3ST3A1	31738491	31739113	19	622	-2.165059246	-6.676566257	hypo
CBFA2T2	63098081	63098880	13	799	-2.18031227	-5.632250761	hypo
THAP2	1306958	1307460	5	502	-2.589132252	-6.803085675	hypo
ZSCAN20	112321921	112323488	3	1567	-1.835351451	-5.337532751	hypo
IFNAR2	2315590	2315960	1	370	-2.265586087	-5.008212583	hypo
HTRA1	42342452	42342900	26	448	-1.03201086	-5.695086326	hypo
MMP1	5820681	5820952	15	271	-2.332310113	-7.30646241	hypo
TNRC18	38909643	38911383	25	1740	-1.166719991	-6.250459085	hypo
PCDHGA2	52511811	52512620	7	809	-2.571379947	-5.853256371	hypo
THAP8	46703161	46703637	18	476	-2.297430562	-7.794981691	hypo
LOC618787	4967401	4969760	7	2359	-1.629801932	-6.346940942	hypo
SAMD9	10451101	10451857	4	756	-1.633401832	-6.859380752	hypo
PCDHGA2	52470921	52471994	7	1073	-2.067535331	-5.873647013	hypo
DUOXA2	65309201	65309514	10	313	-1.849131725	-5.038011075	hypo
CELSR2	34153741	34157401	3	3660	-1.469224046	-6.385267731	hypo
TLCD1	20148861	20149560	19	699	-1.92835789	-5.357622353	hypo
LTBP3	43734503	43735240	29	737	-1.529660798	-5.022776371	hypo
LOC616254	48235186	48235992	19	806	-8.727920455	-8.42802011	hypo
SPOCK1	48366781	48367233	7	452	-3.547900406	-7.819764125	hypo
FAT2	62774561	62775341	7	780	-3.077485471	-5.492806294	hypo
P2RY2	52723080	52724900	15	1820	-2.701815211	-5.909246176	hypo
FAM20C	42135203	42136026	25	823	-2.052929886	-5.536752322	hypo
VWC2	5990776	5991340	4	564	-4.267823683	-7.859661945	hypo
DYNAP	54013521	54013960	24	439	-2.614924113	-7.560036345	hypo
PATZ1	70142261	70143759	17	1498	-3.100417051	-5.116339635	hypo
BMP7	58889621	58890520	13	899	-1.68856408	-6.552109955	hypo
ZNF518A	17503981	17506000	26	2019	-1.188250992	-5.706414836	hypo
JADE2	46100346	46100811	7	465	-1.532631548	-6.574760846	hypo
PHF20	65011906	65012291	13	385	-1.065103992	-6.331167496	hypo
SETMAR	22017101	22018264	22	1163	-3.395798804	-7.625623899	hypo
NLRX1	29756069	29756885	15	816	-1.110711373	-5.732308157	hypo
APC2	43874981	43877340	7	2359	-2.057769594	-5.01278269	hypo
PSMB8	7173061	7173360	23	299	-1.787689727	-5.723485462	hypo
SETD1B	53492651	53493890	17	1239	-1.398408551	-5.207410366	hypo
PHLDB1	29310061	29310613	15	552	-1.088433447	-5.211328793	hypo
LOC101904962	20385256	20386119	12	863	-2.588397176	-6.791869102	hypo
ROBO1	26157756	26158092	1	336	-1.933909042	-5.389539415	hypo
C2H2orf88	6103941	6104600	2	659	-2.845406968	-5.989847885	hypo
FRMD4B	32381001	32381280	22	279	-2.317290132	-7.205349826	hypo
FAT2	62772073	62773080	7	1007	-2.637102662	-5.110919848	hypo
SPPL3	63027921	63028512	17	591	-1.269196612	-5.946933263	hypo
TSNARE1	1976638	1977073	14	435	-3.335390355	-6.77883175	hypo
UBTD2	3997790	3998381	20	591	-1.422826301	-5.930254005	hypo

ZNF362	120425925	120426150	2	225	-3.141958145	-7.259351341	hypo
PCDHGA2	52425021	52426520	7	1499	-2.979997655	-6.026349224	hypo
LOC104973427	73946821	73947113	11	292	-1.231449229	-5.160989978	hypo
ZSCAN29	55121154	55121590	21	436	-1.348060453	-7.139571417	hypo
NUPR1	26082045	26082342	25	297	-1.830591289	-6.982465061	hypo
TGFB2	21808221	21808712	16	491	-1.401904827	-6.635532506	hypo
PCDHGA2	52464261	52466780	7	2519	-1.538321776	-5.168167584	hypo
TLR4	107065850	107066700	8	850	-2.571382412	-6.11543578	hypo
TBCEL	31648678	31649180	15	502	-2.6143917	-6.597941998	hypo
SCN1B	45780781	45781391	18	610	-1.778168084	-7.014828437	hypo
ABO	104176829	104177300	11	471	-2.908800186	-6.326614518	hypo
PHACTR4	124947253	124947649	2	396	-1.1351186	-5.60270989	hypo
JAM3	32993965	32994720	29	755	-1.430978083	-5.362047185	hypo
SMTNL2	25112573	25113046	19	473	-1.287417495	-7.011416004	hypo
LOC508131	59603261	59604600	18	1339	-1.620796963	-7.295700934	hypo
LOC509034	86903301	86904300	10	999	-1.492046533	-5.519390137	hypo
ZFP30	47616401	47617640	18	1239	-2.545722605	-6.239388813	hypo
NCOA5	74805344	74805671	13	327	-1.737168206	-5.182514005	hypo
D2HGDH	120790672	120791380	3	708	-1.984050794	-6.710123812	hypo
DOLK	99443607	99445560	11	1953	-1.642616254	-5.228015913	hypo
USP30	63983619	63984400	17	781	-1.635302389	-6.91492258	hypo
SLC16A5	56338721	56339873	19	1152	-1.862138197	-5.103635323	hypo
PLCD3	44781285	44781916	19	631	-1.78137277	-5.033272295	hypo
MYORG	75742781	75743920	8	1139	-2.972632593	-7.705401592	hypo
LYSMD4	6826001	6826640	21	639	-2.280391245	-5.039114905	hypo
NUDT18	69420700	69421720	8	1020	-1.950545307	-7.388820411	hypo
SIX5	53308670	53309473	18	803	-2.31307753	-5.098338642	hypo
IQCN	4930261	4931722	7	1461	-1.445463595	-6.191489788	hypo
PPFIBP2	45065945	45066652	15	707	-2.633473805	-5.428986655	hypo
TNF	27716701	27717280	23	579	-6.47648719	-7.618041013	hypo
PCDHB8	52295921	52296740	7	819	-2.170349573	-6.241632773	hypo
ZBED5	41821101	41821540	15	439	-1.238389521	-5.335661051	hypo
PCDHB11	52339981	52341580	7	1599	-2.807203704	-5.858312934	hypo
PNPLA6	16399808	16400764	7	956	-1.916888408	-5.11470339	hypo
MBTPS2	121447841	121448660	30	819	-1.723106026	-5.606668828	hypo
EXOC3L2	52898341	52898847	18	506	-7.920055055	-7.124749031	hypo
ELOVL6	15358119	15358780	6	661	-1.339297119	-5.188937999	hypo
LEO1	58439976	58440220	10	244	-2.045674904	-5.50133418	hypo
MED18	125068741	125069534	2	793	-1.076970234	-5.290222686	hypo
LOC100140915	63803123	63805300	18	2177	-1.592702969	-6.722283238	hypo
ZBTB7A	19975401	19976865	7	1464	-1.208550468	-5.404979051	hypo
GALNT7	5591750	5591959	8	209	-1.13770433	-5.999903247	hypo
NOTCH3	7931023	7931356	7	333	-2.579226131	-5.850272683	hypo
STIM2	46454460	46454860	6	400	-1.402540438	-5.02169793	hypo
FAM83F	111390965	111392558	5	1593	-1.664425037	-5.321402416	hypo
LRIF1	32421741	32422260	3	519	-1.600642923	-5.43800494	hypo
ZNF407	3571881	3573080	24	1199	-1.108857545	-5.268614236	hypo
RGL2	7465580	7465800	23	220	-1.602280636	-5.556234669	hypo
STRBP	94273661	94274340	11	679	-2.457373453	-5.977728206	hypo
ZADH2	3492266	3493320	24	1054	-1.265691953	-6.095754406	hypo
PPP1R14A	48069556	48069952	18	396	-1.332187737	-5.138197175	hypo
RASSF10	39680341	39682060	15	1719	-3.567568238	-5.375111174	hypo
SWT1	66273161	66274077	16	916	-2.431958439	-6.124735024	hypo
LOC112447087	79182641	79182962	6	321	-3.587215183	-5.6527049	hypo
SLC43A2	22691001	22692199	19	1198	-3.833432718	-5.80960987	hypo
LOC509034	86904341	86904660	10	319	-1.313584636	-5.158189335	hypo
ZFYVE1	84486397	84487520	10	1123	-1.230789075	-7.136606247	hypo
DHX34	54347361	54348354	18	993	-1.344028397	-5.27064442	hypo

AMER1	96146401	96147260	30	859	-1.536982472	-5.478612416	hypo
YPEL1	72081101	72082440	17	1339	-1.850903223	-5.537725286	hypo
PCLO	37476302	37477660	4	1358	-1.249387622	-6.352684732	hypo
GALNT5	39067668	39069640	2	1972	-3.895901274	-5.438170237	hypo
RASSF6	88624116	88624580	6	464	-2.223987147	-7.29722425	hypo
LOC786489	52259401	52260020	7	619	-3.207800835	-5.730894652	hypo
RASA1	87039001	87039260	7	259	-2.842436031	-5.775307679	hypo
PCDH18	20300135	20300940	17	805	-2.062386641	-7.328949608	hypo
PYGO1	54831741	54832680	10	939	-1.223747701	-6.026231618	hypo
LOC787102	52291241	52292340	7	1099	-1.861565051	-6.028726268	hypo
RPS6KC1	70349441	70350163	16	722	-1.421194308	-6.548863501	hypo
AGGF1	8202183	8202514	10	331	-1.009944465	-6.869474894	hypo
FRMPD1	61863921	61865028	8	1107	-3.088471445	-7.879121141	hypo
ATCAY	20079481	20080588	7	1107	-1.784298854	-7.032299037	hypo
LCOR	18252001	18253980	26	1979	-1.011484014	-6.253779945	hypo
ZNF410	85319301	85319580	10	279	-1.241602695	-5.254561505	hypo
RPGR	104982661	104983554	30	893	-1.305990553	-7.234684069	hypo
MAP1S	5408683	5408917	7	234	-1.894415368	-5.98387323	hypo
PDGFC	42478159	42478540	17	381	-2.074071042	-6.822452859	hypo
SERPINB6	50750581	50750964	23	383	-3.60593663	-6.314192298	hypo
ID3	129297874	129298160	2	286	-1.368495507	-6.571732579	hypo
LPAR6	18204041	18204700	12	659	-1.830777331	-6.8503753	hypo
TANC1	37134921	37136403	2	1482	-1.161088871	-6.019737795	hypo
ZFHX2	21410453	21412480	10	2027	-2.311175039	-7.218597919	hypo
FLRT2	97153541	97154204	10	663	-1.640831801	-6.889101357	hypo
MYH15	53144341	53144640	1	299	-2.544212465	-6.978188233	hypo
CDKN2B	22086721	22087035	8	314	-1.364103616	-5.350643139	hypo
NFKB2	23025812	23026096	26	284	-1.106264303	-6.642506766	hypo
FAM122A	44894737	44895472	8	735	-1.343123205	-6.151387615	hypo
EXT2	74352266	74353120	15	854	-1.082714266	-5.557764763	hypo
ANGPT4	60234003	60234780	13	777	-8.613973587	-7.877567048	hypo
ZFHX2	21420021	21421920	10	1899	-2.404862102	-5.491985286	hypo
FAT2	62749681	62750500	7	819	-3.771364438	-5.445813867	hypo
GLYR1	3883921	3884767	25	846	-1.21570401	-6.077511528	hypo
TUBB4A	17987161	17987960	7	799	-1.24038767	-6.292669107	hypo
RNF150	16867251	16867462	17	211	-1.196950634	-6.333621187	hypo
MYO9A	18677041	18677814	10	773	-1.434410081	-6.054593335	hypo
SLIT2	39779281	39780073	6	792	-1.22152001	-7.309034243	hypo
CSF1	33495887	33496909	3	1022	-1.72377847	-5.000517837	hypo
KIF3C	73493023	73494260	11	1237	-1.207554984	-6.480338651	hypo
ZNF827	12577381	12578540	17	1159	-3.57133316	-7.441068528	hypo
TIMP1	85943284	85943558	30	274	-1.287346031	-5.479796662	hypo
SNX29	11277681	11278340	25	659	-3.319513542	-5.075526713	hypo
EFNA4	15532625	15533332	3	707	-1.4962763	-6.693966451	hypo
GLI3	79059610	79062140	4	2530	-1.191996867	-6.500339162	hypo
OR9Q2	81371461	81372540	15	1079	-5.350238491	-7.872097327	hypo
SIK3	27527545	27527885	15	340	-1.651107503	-6.601218114	hypo
SLC43A2	22687301	22687660	19	359	-4.522339506	-7.480768196	hypo
KEAP1	15009367	15010129	7	762	-1.06517616	-6.912102256	hypo
LRFN3	46632141	46633581	18	1440	-1.814419101	-5.072022337	hypo
ERCC6L2	82671470	82672000	8	530	-1.877300709	-5.89636694	hypo
NCOA5	74798993	74799337	13	344	-2.151764217	-6.72672207	hypo
MINDY2	51427521	51428180	10	659	-2.063380201	-5.311124378	hypo
JADE2	46115941	46116600	7	659	-3.154650312	-5.850589502	hypo
PCDHGA2	52442486	52443060	7	574	-2.904688728	-6.713229367	hypo
MGC139164	60856341	60856760	18	419	-2.679261526	-6.273788248	hypo
LOC532875	14765081	14766120	18	1039	-1.100683334	-6.456064016	hypo
LOC101908039	22564341	22564800	8	459	-2.430373428	-5.662349414	hypo

ZNF830	15119021	15120467	19	1446	-1.433368429	-6.431107031	hypo
TMEM191C	72207601	72208529	17	928	-1.202111857	-5.166994162	hypo
INHBB	72112661	72113180	2	519	-1.553572082	-5.409485546	hypo
DLG3	79948621	79949270	30	649	-1.883723542	-5.891726187	hypo
GPR153	47099213	47100280	16	1067	-1.756313677	-5.38932923	hypo
RPUSD2	36170913	36171920	10	1007	-1.260529623	-7.640198348	hypo
SPRED3	48204021	48205120	18	1099	-1.958457738	-5.465655773	hypo
CCDC60	55895961	55896187	17	226	-2.96211524	-5.986368948	hypo
NKPD1	52806782	52808887	18	2105	-3.024993152	-5.146191903	hypo
LOC100336476	119472902	119474789	3	1887	-1.854636555	-5.145205449	hypo
AP2M1	82970226	82970700	1	474	-1.023084696	-5.916195328	hypo
TUBA4A	107200121	107200480	2	359	-1.315975223	-6.684450943	hypo
TNRC18	38923070	38923500	25	430	-1.266778147	-6.78866265	hypo
TMEM200A	68454681	68455065	9	384	-2.402591926	-5.460671654	hypo
KRT13	41709890	41710111	19	221	-4.155231931	-5.414431619	hypo
PCDHGA2	52475421	52477308	7	1887	-2.422778031	-5.407114581	hypo
LRIF1	32422621	32422992	3	371	-1.79531659	-6.983175085	hypo
CHAMP1	87093771	87094760	12	989	-1.413052	-6.328587726	hypo
GPRIN2	41806021	41806760	28	739	-2.598602177	-5.382240411	hypo
STRC	55328392	55328600	21	208	-2.212250411	-7.258847465	hypo
TCF25	14701503	14702859	18	1356	-1.04043754	-5.821068414	hypo
VCPIP1	30874681	30875540	14	859	-1.036818809	-6.57334592	hypo
PHTF1	29706942	29707440	3	498	-3.187214911	-7.137985004	hypo
SNX21	74603372	74603718	13	346	-1.726104602	-5.174126345	hypo
TRAF1	110293205	110294512	8	1307	-1.817793374	-5.802746311	hypo
PCDHGC3	52566741	52567720	7	979	-7.965207091	-6.958643647	hypo
MOSMO	19764263	19764850	25	587	-2.155423154	-6.083387851	hypo
DHX34	54344441	54345193	18	752	-1.116312686	-7.532409377	hypo
CARD6	33637447	33637996	20	549	-3.563168344	-5.457218937	hypo
SEMA4G	21820741	21821593	26	852	-1.323220125	-5.009570563	hypo
TRIM21	50647717	50648540	15	823	-1.246467651	-7.565639482	hypo
GLCE	16131996	16132242	10	246	-1.775243957	-5.921737609	hypo
NFE2L3	69781993	69782640	4	647	-2.797915226	-5.057419325	hypo
LOC112441510	41825621	41825842	19	221	-2.153122697	-6.151427734	hypo
PTPN9	33375361	33375780	21	419	-2.634775467	-6.242799973	hypo
ADAMTS6	14330174	14330620	20	446	-1.5325465	-5.054936536	hypo
CLDN1	76988870	76989200	1	330	-1.390998824	-7.003869027	hypo
MUC1	15428467	15429801	3	1334	-2.655186216	-5.352974387	hypo
SMAD1	12741781	12742156	17	375	-1.436372717	-6.832917269	hypo
RIN1	44383561	44384582	29	1021	-1.432959407	-7.083676092	hypo
UVSSA	117094321	117094780	6	459	-1.122105588	-5.294532087	hypo
IDUA	117373047	117374376	6	1329	-1.934736069	-6.684037536	hypo
ZNF398	112279369	112280860	4	1491	-1.463654858	-5.538802998	hypo
PCDH1	52948775	52949638	7	863	-1.177985047	-6.281373935	hypo
CRBN	23146499	23147060	22	561	-1.559709958	-5.591665022	hypo
SH3PXD2A	24166221	24166800	26	579	-1.694586992	-5.769384705	hypo
N4BP2L2	28577468	28578140	12	672	-1.051988925	-5.432997526	hypo
MYH15	53174438	53174828	1	390	-2.928074779	-5.167281852	hypo
SALL2	25652541	25653800	10	1259	-2.50385945	-6.766900007	hypo
TP63	77479319	77479574	1	255	-1.703251754	-5.665424125	hypo
ASTE1	138840061	138841064	1	1003	-1.287415803	-6.31929049	hypo
UBR2	16361542	16362300	23	758	-1.05422618	-6.480541112	hypo
YPEL1	72080615	72080980	17	365	-3.479142045	-6.060052857	hypo
TPGS1	43164861	43165361	7	500	-1.309272352	-6.249598666	hypo
NAT1	39014461	39015140	27	679	-3.273164805	-5.942142347	hypo
PDE4B	78958721	78959471	3	750	-1.704206145	-6.850013239	hypo
TMEM203	106223225	106224140	11	915	-1.220738803	-5.857285863	hypo
RPP38	29679318	29680560	13	1242	-1.220195609	-7.341833718	hypo

TNRC18	38944607	38944850	25	243	-1.013385563	-6.706896692	hypo
KMT2E	46268266	46268531	4	265	-1.245480708	-6.931166998	hypo
CERK	116906780	116908480	5	1700	-1.134931895	-6.330540303	hypo
PCF11	12495140	12495580	29	440	-2.141962648	-6.660407555	hypo
DCUN1D1	84074647	84075140	1	493	-1.074866306	-6.69501786	hypo
BMF	35754174	35754471	10	297	-2.062622043	-5.287332853	hypo
AIF1	27685442	27685653	23	211	-3.157721968	-5.683351699	hypo
C10H14orf93	21849321	21850363	10	1042	-1.656517875	-5.289669241	hypo
C23H6orf47	27641181	27641451	23	270	-2.608571128	-5.41183942	hypo
HARBI1	76569121	76569720	15	599	-1.489270886	-5.042152307	hypo
CCDC191	58715859	58716083	1	224	-1.116017654	-5.208167561	hypo
PROCA1	20131021	20131814	19	793	-1.612099049	-7.13827839	hypo
WDR81	22826813	22827503	19	690	-1.695734634	-6.954093232	hypo
FOXP2	53771701	53772116	4	415	-2.119424929	-5.088390083	hypo
ROBO1	26493566	26493893	1	327	-2.072586047	-5.597613001	hypo
SLC25A29	65203741	65205094	21	1353	-1.566469981	-6.922222006	hypo
CYP1B1	20471181	20471796	11	615	-3.550293116	-5.882735166	hypo
TMEM79	14513891	14514620	3	729	-1.604962715	-6.418374982	hypo
KLHDC8A	3105228	3105788	16	560	-3.057794074	-5.268634957	hypo
SHROOM4	88412141	88412480	30	339	-1.689093653	-7.100232804	hypo
GOLGA3	44454979	44455243	17	264	-1.636391302	-6.593275748	hypo
EBAG9	54843681	54844133	14	452	-1.226397947	-5.123624426	hypo
C4H7orf25	78145941	78146860	4	919	-2.939291119	-5.341568435	hypo
CHST14	36077443	36078860	10	1417	-1.630420676	-6.606280203	hypo
CLIP2	33056241	33057965	25	1724	-1.411821683	-6.276996209	hypo
ADAL	55115708	55116360	21	652	-2.364147682	-5.492116711	hypo
ZBTB37	55240319	55241279	16	960	-1.716761323	-6.146847502	hypo
GTPBP6	88349645	88350500	1	855	-1.765397641	-6.005846484	hypo
PCDHGA2	52484221	52484700	7	479	-3.293932147	-5.113289682	hypo
B4GALNT3	107426799	107427604	5	805	-2.51470301	-5.642525922	hypo
ESCO1	34643761	34644180	24	419	-1.008217162	-5.61081655	hypo
ZMIZ1	34943381	34944480	28	1099	-1.109646666	-5.881933277	hypo
LOC100336476	119448494	119449494	3	1000	-3.002659008	-7.325652253	hypo
TRIM45	26100401	26100886	3	485	-2.092495968	-5.44091962	hypo
ZBTB4	27134361	27136351	19	1990	-1.344386806	-6.039639081	hypo
FAM198B	40796022	40797166	17	1144	-8.433376793	-8.013907277	hypo
STK36	106658993	106659540	2	547	-1.812731256	-5.08637487	hypo
GJD3	40604665	40605360	19	695	-5.101194262	-7.498581727	hypo
GPR132	69438921	69439920	21	999	-1.953831906	-7.895354508	hypo
ZADH2	3493621	3494640	24	1019	-1.065776843	-6.61071721	hypo
AKT3	33633372	33633720	16	348	-1.037754067	-6.425189091	hypo
RALGAPA1	45883181	45883560	21	379	-2.242124486	-6.05411965	hypo
MAN2A2	21739541	21741400	21	1859	-1.442280664	-6.564547744	hypo
FHOD1	34861176	34861787	18	611	-1.508027462	-5.665395994	hypo
ZSCAN20	112326941	112327625	3	684	-1.972670827	-7.399090713	hypo
SALL2	25650788	25652160	10	1372	-3.003531691	-7.208897211	hypo
ANGPTL2	97857492	97858358	11	866	-1.492573654	-6.181435472	hypo
TACR2	25769941	25770440	28	499	-4.063734952	-7.942646126	hypo
BDH1	71964601	71965496	1	895	-1.114067394	-5.447064282	hypo
MARVELD1	18871861	18872335	26	474	-1.444306373	-5.819270701	hypo
ZBTB22	7484861	7486196	23	1335	-1.316406184	-6.268241573	hypo
TRANK1	10682301	10682720	22	419	-3.893311446	-6.652846044	hypo
CAPN14	68742699	68743160	11	461	-1.841192657	-7.593869166	hypo
PCLO	37684669	37685520	4	851	-2.969704375	-5.273516644	hypo
MCIDAS	23913556	23914260	20	704	-1.657622995	-5.031516704	hypo
ATP23	55516049	55516511	5	462	-1.69676406	-6.430080514	hypo
TP53BP1	55175633	55176064	21	431	-1.655073049	-5.459025891	hypo
ZC3H4	54112961	54113660	18	699	-1.352214843	-5.039671571	hypo

SIX5	53305387	53306654	18	1267	-2.818433366	-5.079744069	hypo
ZNF182	86423601	86424708	30	1107	-1.991086034	-5.10169936	hypo
SUPT7L	72041499	72042260	11	761	-1.08514756	-5.073664148	hypo
INTS5	40982521	40985320	29	2799	-1.48077281	-5.654551656	hypo
AMER1	96142061	96143400	30	1339	-1.050570793	-6.17524855	hypo
ZDHHC8	73011661	73012626	17	965	-1.244288129	-6.566728956	hypo
APC	1173061	1173320	10	259	-1.094070946	-6.320642356	hypo
CABIN1	71414149	71414396	17	247	-1.361888288	-5.094377842	hypo
ZBTB4	27131981	27133020	19	1039	-1.786193996	-6.800243593	hypo
DBP	55275373	55275585	18	212	-1.307119818	-5.167211635	hypo
ZNF268	44224381	44225400	17	1019	-4.015483157	-6.067851427	hypo
SMAGP	28546341	28546720	5	379	-2.031487301	-5.073829018	hypo
SNX19	36942604	36943660	29	1056	-1.684354844	-6.903317661	hypo
VEZF1	8777581	8778043	19	462	-1.104203476	-6.262974369	hypo
LOC101903326	1089721	1090900	14	1179	-8.185866545	-8.599141382	hypo
LRIG3	54611526	54612140	5	614	-1.598882078	-7.424563042	hypo
SYNPO	61990227	61990886	7	659	-7.712870868	-5.179964979	hypo
LRP8	92996821	92997920	3	1099	-1.911562486	-6.641337651	hypo
PARD6G	417041	417580	24	539	-2.865413077	-6.26844025	hypo
ERCC6L2	82710593	82711080	8	487	-2.420857534	-5.712036444	hypo
GOLGA3	44439887	44440546	17	659	-1.286830145	-5.32603928	hypo
HOXA10	68870601	68871860	4	1259	-1.655883231	-5.61945221	hypo
TOM1	73588341	73588704	5	363	-1.300509111	-5.094223376	hypo
FAM83H	1033018	1033480	14	462	-1.451452895	-6.577720484	hypo
SAA3	26414395	26414676	29	281	-4.678938649	-7.870458484	hypo
FER	108280477	108281780	7	1303	-1.026604427	-5.173130883	hypo
EBP	86661606	86662086	30	480	-1.579874296	-5.01099967	hypo
ZNF286A	33083301	33083940	19	639	-1.513041649	-5.259754432	hypo
FLYWCH1	2364501	2366051	25	1550	-3.229419688	-5.229570119	hypo
ISLR	34510341	34511691	21	1350	-4.065250008	-5.661522121	hypo
TCF12	53036100	53036333	10	233	-1.362933767	-5.108790084	hypo
PREP	44707851	44708317	9	466	-1.138996931	-7.693775959	hypo
FAM110A	60259693	60261216	13	1523	-1.312314515	-7.277462516	hypo
ROBO1	26125640	26125949	1	309	-2.503694639	-5.158950942	hypo
TLDC1	10689521	10690146	18	625	-1.168173077	-6.88389249	hypo
EXOSC6	1814581	1815582	18	1001	-1.331880113	-5.678944267	hypo
PCDHB14	52329821	52330880	7	1059	-2.477201141	-7.378733712	hypo
CREB3L2	101604041	101604660	4	619	-2.29406147	-5.055001021	hypo
RUFY1	1706055	1706660	7	605	-1.111399641	-5.247035608	hypo
SESN3	15268541	15268880	15	339	-2.356660364	-5.320770334	hypo
ZSCAN29	55110901	55111660	21	759	-1.828537889	-5.200651757	hypo
ZC3H4	54135238	54135458	18	220	-1.032562344	-6.268631946	hypo
PPP2R3A	133118641	133119340	1	699	-2.314378754	-5.185925822	hypo
LOC104975635	3168181	3168640	23	459	-1.700666183	-5.251204909	hypo
PARP9	66978801	66979400	1	599	-3.490769049	-6.68289183	hypo
ZC3H4	54113761	54115489	18	1728	-2.039994088	-5.641858331	hypo
PASK	120343486	120343686	3	200	-3.360518757	-6.984099157	hypo
NOL8	83941901	83942600	8	699	-1.935988792	-7.579176722	hypo
KIAA1324	34199841	34200300	3	459	-2.222783449	-5.378593865	hypo
ARHGAP26	54278658	54279120	7	462	-2.896007579	-7.093588093	hypo
ZNF469	13699381	13703480	18	4099	-2.297107969	-5.155458227	hypo
BTBD8	51230121	51230808	3	687	-1.808336949	-5.56788591	hypo
NCOA1	74447901	74448580	11	679	-1.011398005	-5.328092565	hypo
BORCS8	4077657	4077983	7	326	-1.681221652	-6.063166433	hypo
GSTT4	71311337	71311952	17	615	-1.987698405	-5.537218365	hypo
KCNH3	30274162	30275134	5	972	-3.098711765	-5.783863863	hypo
MOSPD3	35950130	35950348	25	218	-2.111591691	-6.453927105	hypo
PRKCQ	17098880	17099700	13	820	-1.411434089	-5.013256633	hypo

MTAP	22191061	22191416	8	355	-1.165928246	-5.155670913	hypo
DHRS13	20269721	20270298	19	577	-1.318747286	-5.056719268	hypo
EIF4G3	131836841	131837400	2	559	-1.581789998	-5.455774094	hypo
ATP7B	21342221	21343194	12	973	-2.13195548	-5.470121962	hypo
MORN4	18795096	18795580	26	484	-2.404251078	-6.506849979	hypo
MALT1	57838779	57839540	24	761	-1.58516742	-7.189160602	hypo
ZMIZ1	34941312	34942940	28	1628	-1.26629772	-5.950900671	hypo
SHROOM4	88426319	88427132	30	813	-1.983709344	-7.264660404	hypo
EVPL	55642157	55644365	19	2208	-2.606017106	-5.012960038	hypo
DHX34	54370900	54372020	18	1120	-2.559023101	-7.534269074	hypo
PARP9	66966584	66966860	1	276	-3.20056893	-6.478738288	hypo
DSEL	9447521	9450420	24	2899	-1.49354643	-7.058364706	hypo
TNRC18	38936514	38936875	25	361	-1.172395684	-6.239376024	hypo
GGTA1	92318021	92318723	11	702	-1.61484417	-6.632742346	hypo
IGSF3	26701055	26701306	3	251	-1.273120142	-6.884857876	hypo
SIK3	27513321	27513760	15	439	-2.036558545	-6.282958397	hypo
ZNF189	91129321	91129580	8	259	-1.853893961	-7.482410857	hypo
FUT1	55379101	55380140	18	1039	-2.311475459	-5.472070463	hypo
NLGN1	93147021	93147982	1	961	-2.706693377	-5.57944571	hypo
MARCH9	55706381	55707220	5	839	-1.099526895	-6.676505776	hypo
MC1R	14705621	14706843	18	1222	-3.119239136	-5.773532998	hypo
SIPA1L1	83203921	83204419	10	498	-1.459806296	-6.603947491	hypo
FAM83E	55258041	55258520	18	479	-2.219104474	-7.699361294	hypo
LOC101906012	94261761	94262500	3	739	-1.505653099	-5.287175351	hypo
RUSC1	15308641	15309400	3	759	-2.328773636	-5.319044925	hypo
ARAP2	55608561	55608853	6	292	-1.340539298	-5.471956028	hypo
SHISA7	62076261	62077587	18	1326	-1.33432894	-5.55257228	hypo
ZNF449	18980027	18980914	30	887	-1.282926923	-5.981374684	hypo
MUC4	70483861	70484450	1	589	-7.793765857	-6.702189312	hypo
LOC112445746	11309	11703	NW_020 191998.1	394	-3.547019007	-7.50479626	hypo
CYS1	87542501	87543400	11	899	-3.6508429	-5.738182438	hypo
ZKSCAN2	22823901	22824936	25	1035	-2.827649077	-6.000402788	hypo
FBN1	61917881	61918380	10	499	-4.493933407	-7.753869286	hypo
TTC3	149402721	149403200	1	479	-1.542078288	-6.761627839	hypo
TTLL5	87024395	87024702	10	307	-1.506425667	-6.763822302	hypo
SPG11	102989879	102990633	10	754	-1.270277873	-5.984717378	hypo
RABEP1	26176717	26177185	19	468	-1.062198585	-5.015097962	hypo
PCLO	37659401	37660545	4	1144	-2.115771768	-5.126500775	hypo
LOC532048	60808150	60808400	18	250	-2.286226125	-6.596751884	hypo
FICD	64408381	64409320	17	939	-1.756610123	-5.526420361	hypo
KIF1B	43255541	43256100	16	559	-1.332595922	-5.456252775	hypo
ZNF200	2679801	2681166	25	1365	-2.44839975	-5.010121028	hypo
NRP2	94123960	94124440	2	480	-1.338587787	-6.174464016	hypo
LOC516355	102950131	102950380	4	249	-1.026208058	-6.606666983	hypo
LZTS2	21838972	21839635	26	663	-1.965097041	-6.378612814	hypo
LOC100297097	17958992	17959460	13	468	-2.025344438	-7.397768334	hypo
ARID1B	93840889	93842840	9	1951	-1.051911681	-5.236540253	hypo
LOC787287	63817742	63818800	18	1058	-1.729810729	-6.978085917	hypo
SETD1B	53483054	53484311	17	1257	-1.161364004	-6.215453633	hypo
TNS2	26907401	26908263	5	862	-1.453478576	-5.63650122	hypo
PGGHG	82957138	82957422	11	284	-1.504988347	-5.124377735	hypo
SZT2	102575891	102576600	3	709	-1.421309873	-7.507527612	hypo
CD70	17908292	17908687	7	395	-4.123315033	-5.401425235	hypo
ARSI	61646441	61648420	7	1979	-2.542125141	-5.602169892	hypo
MICALL2	41472809	41473553	25	744	-1.758222199	-6.557991593	hypo
PRPF38A	93935321	93935880	3	559	-1.392711413	-6.1382219	hypo
FAM167A	7911590	7912388	8	798	-3.163299525	-5.152518678	hypo

LYN	23243338	23244380	14	1042	-1.037069536	-7.082967537	hypo
HTRA2	10107232	10107589	11	357	-1.60384472	-7.087516202	hypo
PRR14L	70364021	70364753	17	732	-1.080747414	-6.649752678	hypo
WDR81	22819753	22822880	19	3127	-1.353419518	-6.169329856	hypo
SAP30L	65733201	65733920	7	719	-1.491651165	-5.533731948	hypo
PGGHG	82961790	82963240	11	1450	-1.926095737	-6.176295862	hypo
ETV6	97960096	97960642	5	546	-1.778991683	-7.276602446	hypo
TFCP2	28610703	28611380	5	677	-1.443032035	-5.00002487	hypo
WNT5A	45560361	45560680	22	319	-3.367045188	-6.019425277	hypo
LOC100848077	59524621	59525500	18	879	-1.321365896	-5.841489372	hypo
PCDHB11	52338941	52339920	7	979	-2.547988905	-5.332091248	hypo
RHOBTB1	16652278	16653200	28	922	-2.572190438	-7.080016995	hypo
ZNF845	60674241	60675640	18	1399	-1.756258124	-6.813532615	hypo
EPB41	124429501	124430620	2	1119	-1.049811655	-5.067542585	hypo
CCDC174	57604753	57605500	22	747	-1.86822097	-5.190196033	hypo
WDFY3	99508341	99508755	6	414	-1.772911405	-7.636343146	hypo
MPV17	72334141	72334888	11	747	-1.701597941	-5.354818874	hypo
PPFIBP2	45058386	45059040	15	654	-3.390667686	-7.202454024	hypo
ZFHX2	21422357	21424370	10	2013	-2.388820976	-5.314614755	hypo
PLPP6	39863786	39864900	8	1114	-1.000571176	-5.130748453	hypo
CPLANE1	37096128	37096899	20	771	-1.053174629	-5.424759079	hypo
EXT1	46477327	46477877	14	550	-1.038692741	-6.069890453	hypo
FRMPD1	61862721	61863900	8	1179	-1.805209744	-5.279038399	hypo
STX1A	33560661	33561036	25	375	-1.26120022	-5.480237521	hypo
FOCAD	23422421	23422820	8	399	-1.919045596	-5.482705627	hypo
KLHL9	22874801	22876560	8	1759	-1.56027277	-6.033022278	hypo
WDR81	22830569	22831945	19	1376	-1.345204477	-5.086579453	hypo
ZC3HAV1	102679043	102679380	4	337	-1.584962501	-5.536852441	hypo
SAMD12	46065686	46065920	14	234	-1.477282582	-5.128218775	hypo
ZNF789	36878521	36879300	25	779	-1.975044053	-6.91604302	hypo
ZMYM4	110326881	110327740	3	859	-3.977457431	-7.64098269	hypo
SETDB1	19805609	19806238	3	629	-1.380887624	-6.312945753	hypo
SIK3	27511921	27512400	15	479	-2.206272756	-5.098118791	hypo
KBTBD4	77375279	77375951	15	672	-1.098985145	-6.575658002	hypo
ZSCAN2	22287721	22288820	21	1099	-1.369543535	-5.260220049	hypo
PLD1	95908536	95909140	1	604	-1.785948004	-6.680327852	hypo
NFKB1	22206181	22206821	6	640	-1.834379338	-5.113104831	hypo
C2H2orf88	6102081	6103720	2	1639	-1.867005372	-5.36464447	hypo
PARD6G	415912	417000	24	1088	-1.250347307	-5.683975273	hypo
PTPN22	29558841	29559607	3	766	-2.013840351	-5.278299193	hypo
TMEM185B	71939541	71940200	2	659	-1.721851783	-6.880071341	hypo
ERCC5	79094828	79095900	12	1072	-1.757242245	-6.75822574	hypo
CC2D1B	94016481	94017100	3	619	-1.387587521	-6.074239035	hypo
NFIX	12481181	12482481	7	1300	-1.838097261	-6.667350315	hypo
MLLT3	24068581	24069260	8	679	-1.725403308	-5.359236354	hypo
CMTR2	39767901	39768340	18	439	-1.421534643	-5.238920956	hypo
KIAA1614	62071241	62072515	16	1274	-1.754665268	-6.111295032	hypo
VIRMA	69815521	69815927	14	406	-1.310846634	-5.334324909	hypo
FAM208A	44393695	44394060	22	365	-1.939147035	-7.408142407	hypo
HS3ST3B1	32264651	32265270	19	619	-2.004668308	-6.458992835	hypo
YLPM1	86060450	86060871	10	421	-1.110183783	-6.093068895	hypo
ZNF132	65693781	65694455	18	674	-1.752457237	-6.394102633	hypo
TNFRSF25	46908824	46909417	16	593	-4.337766688	-7.187647659	hypo
UBP1	7649241	7649991	22	750	-1.041009353	-6.0305962	hypo
ZNF605	43972301	43973780	17	1479	-1.791579769	-7.12691327	hypo
LOC784522	12216821	12218697	26	1876	-2.470528584	-6.361663672	hypo
PCDH18	20290381	20290908	17	527	-1.921015325	-5.2606468	hypo
SHROOM4	88412621	88413580	30	959	-1.849105865	-5.296343782	hypo

LOC101905343	25527701	25528780	2	1079	-1.134142105	-5.941930567	hypo
NAV2	25041774	25042045	29	271	-1.614497715	-5.072313202	hypo
ADPRHL2	109722182	109722992	3	810	-1.127602232	-5.55557217	hypo
SPATS2	30304741	30305269	5	528	-2.119265838	-5.920726315	hypo
MOGS	10165893	10166380	11	487	-1.567416509	-5.071111014	hypo
ASF1A	32129036	32129436	9	400	-1.255036428	-6.456389184	hypo
RCOR3	72057761	72058324	16	563	-1.22610017	-5.927480677	hypo
ZNF713	27636033	27637120	25	1087	-1.796805841	-5.360633762	hypo
VDR	32437828	32438880	5	1052	-1.758546638	-6.831549324	hypo
ZNF22	44581021	44582051	28	1030	-2.160427753	-6.305640573	hypo
CPT2	93033301	93033606	3	305	-1.898002932	-5.591004986	hypo
TET3	10560181	10560900	11	719	-1.094115398	-5.810308735	hypo
CYBA	13886181	13886491	18	310	-1.142162566	-6.698045897	hypo
FAM171A2	44156310	44158316	19	2006	-1.955278762	-7.129590479	hypo
ANGPTL2	97836861	97837389	11	528	-1.841814236	-6.311450621	hypo
BRWD1	139253289	139253960	1	671	-1.293028209	-5.304708877	hypo
ZNF677	60082521	60083120	18	599	-2.145293024	-6.762711992	hypo
OXSM	40008565	40009574	27	1009	-2.882239487	-6.923826387	hypo
ZNFX1	77366692	77367200	13	508	-1.509058724	-6.354033209	hypo
SYNJ1	2915798	2916780	1	982	-1.449840471	-5.047095304	hypo
NHS	125753461	125754271	30	810	-1.510065391	-6.87910208	hypo
FUT8	77837149	77837940	10	791	-1.68395551	-5.45631924	hypo
KIAA1549	102495221	102495859	4	638	-2.546044968	-7.335426076	hypo
INTS3	16663861	16664160	3	299	-1.535817918	-5.16622856	hypo
F8A1	36138941	36140040	30	1099	-1.82812328	-5.593360073	hypo
TCP11L2	69711261	69711600	5	339	-2.456224954	-5.456683889	hypo
ZBTB11	46058301	46059580	1	1279	-1.55140549	-6.166907899	hypo
SRRM3	34334483	34334699	25	216	-4.416848429	-7.685253748	hypo
ZFP2	2442161	2442760	7	599	-2.58601181	-7.07360888	hypo
NUCB1	55503181	55503527	18	346	-1.148524404	-6.431432095	hypo
KIAA1614	62047625	62048572	16	947	-1.902766181	-7.304857106	hypo
PTPRK	66134475	66134778	9	303	-1.228675842	-5.949814846	hypo
KLHL23	26587610	26588100	2	490	-2.192162491	-7.472420669	hypo
PPP1R14D	36376515	36376860	10	345	-4.03087038	-6.178439924	hypo
GPR156	64940401	64941080	1	679	-3.67556505	-6.052307337	hypo
LOC112445429	14096	14660	NW_020 190482.1	564	-1.305793035	-6.146647042	hypo
HECTD4	62005521	62006686	17	1165	-1.413643743	-6.999264807	hypo
ARL2	43234563	43235043	29	480	-2.379667874	-6.949690924	hypo
PLEKHG2	49115882	49116816	18	934	-1.397002154	-6.149973492	hypo
DTX3L	66994784	66995002	1	218	-2.371364934	-6.504368966	hypo
TSPAN1	99895915	99896260	3	345	-2.166326359	-6.874737124	hypo
DTX3L	66993241	66993720	1	479	-3.236090326	-7.504498637	hypo
ATP10D	66270553	66271220	6	667	-1.809520361	-5.79662107	hypo
DCLRE1C	29553801	29555340	13	1539	-1.106832312	-6.978259774	hypo
PCDH12	53008891	53009738	7	847	-1.637173738	-5.079428649	hypo
LOC104970105	2782712	2783520	25	808	-1.054560718	-5.267153268	hypo
DBN1	38954058	38954360	7	302	-1.647320942	-5.128063052	hypo
FZD6	61184381	61184680	14	299	-1.383375207	-6.008190152	hypo
TGFB2	21807923	21808180	16	257	-1.229515678	-6.013815749	hypo
HOXC8	26021041	26021532	5	491	-1.218809245	-6.716562014	hypo
SLC2A4RG	54030865	54031282	13	417	-1.323238835	-6.71844099	hypo
ARID2	34310481	34310919	5	438	-1.82907946	-5.141328181	hypo
GPRIN2	41806801	41807620	28	819	-2.610655368	-6.560140554	hypo
CCDC189	26877781	26878800	25	1019	-2.096187655	-5.589127657	hypo
ADGRF4	20759361	20759820	23	459	-2.68817574	-7.19016824	hypo
VPS13B	64695041	64695553	14	512	-1.211183463	-7.569930341	hypo
INHBA	79299011	79300035	4	1024	-1.056397969	-6.607200373	hypo

SRRM3	34336510	34337240	25	730	-4.967674472	-7.658777766	hypo
NAPEPLD	44436564	44437208	4	644	-1.545951836	-5.222729343	hypo
IRGQ	51818486	51819018	18	532	-1.230959816	-5.797114831	hypo
CYS1	87541766	87542340	11	574	-2.970996042	-6.077337266	hypo
TTC22	91512013	91513040	3	1027	-1.399351141	-6.819324733	hypo
CELSR3	51222421	51224420	22	1999	-2.885878391	-6.101607307	hypo
ST5	43870361	43870820	15	459	-2.000801275	-5.360572106	hypo
PCDHGA2	52598321	52598880	7	559	-2.284203313	-5.443723859	hypo
ZBTB26	94084541	94085840	11	1299	-1.60621178	-5.148198428	hypo
IRGQ	51811817	51812400	18	583	-1.755081046	-7.750878501	hypo
PRAP1	25655737	25656280	26	543	-2.345430215	-5.658133876	hypo
ZBED5	41820501	41820840	15	339	-2.506301415	-6.422712498	hypo
PLEKHG2	49107835	49108696	18	861	-1.119083353	-6.409447584	hypo
LOC104975673	30493866	30494845	23	979	-2.591328011	-6.482814488	hypo
RTL6	115129301	115130186	5	885	-1.046964104	-5.732550572	hypo
TIAM1	4271792	4272766	1	974	-1.210782111	-6.952112098	hypo
KDELC2	18149061	18149489	15	428	-1.989139007	-7.032216943	hypo
ZNF213	2580651	2582195	25	1544	-1.640130783	-5.379290104	hypo
NEK9	86332661	86333213	10	552	-1.286280501	-5.348894324	hypo
NDE1	14127981	14128660	25	679	-3.176308618	-5.663435339	hypo
ZNF329	65518956	65520500	18	1544	-1.314559785	-5.760795727	hypo
TUBA1D	107219456	107220160	2	704	-1.953958959	-5.427249029	hypo
FOXN1	20003597	20004560	19	963	-3.170344024	-7.699819358	hypo
TTC30A	19259701	19261400	2	1699	-2.89736095	-7.318116573	hypo
HOMER3	4283881	4284203	7	322	-1.588767305	-5.153935728	hypo
WDR89	75839121	75839720	10	599	-1.856383258	-5.390394747	hypo
OVOL2	38271781	38272471	13	690	-1.911679323	-7.46834744	hypo
LOC524576	74499515	74500000	5	485	-1.893133856	-5.546550048	hypo
KDF1	126266880	126267951	2	1071	-1.178408665	-6.317115192	hypo
LIF	69261181	69262100	17	919	-1.930467922	-5.106914193	hypo
CREBBP	3055321	3057629	25	2308	-1.060391348	-6.31066176	hypo
DNAJC22	30470980	30471296	5	316	-1.416234686	-5.665826168	hypo
POU6F1	28588992	28589246	5	254	-3.317304068	-6.626699314	hypo
RBM33	117462753	117463240	4	487	-1.444293391	-6.897255794	hypo
SLC10A3	37548361	37549254	30	893	-1.14577129	-6.414725409	hypo
LOC107133032	18206301	18207113	13	812	-2.419450764	-5.163505765	hypo
EGR2	18936461	18938246	28	1785	-1.049677025	-6.979723747	hypo
FAT2	62773421	62774540	7	1119	-2.6990983	-5.20374486	hypo
TCF7	45799283	45800060	7	777	-1.853431218	-7.023210759	hypo
ADAMTS6	14330961	14331260	20	299	-2.746369005	-5.582769332	hypo
ARMC10	44425621	44425940	4	319	-2.48247498	-5.628945801	hypo
SDR42E1	8544881	8545380	18	499	-2.708298267	-6.31592332	hypo
GTF3C2	72320621	72321460	11	839	-1.888060127	-6.929991617	hypo
BAD	42573697	42574197	29	500	-1.350182716	-7.314896082	hypo
LOC510362	97042301	97043940	30	1639	-1.383650611	-6.42365898	hypo
FAM21A	44155081	44155387	28	306	-2.118298153	-5.461863388	hypo
FUT1	55377841	55378840	18	999	-2.216382738	-5.033724766	hypo
ZNF792	45697061	45697680	18	619	-2.406132115	-6.324613745	hypo
LOC104975635	3169341	3170140	23	799	-1.373600948	-6.046165067	hypo
KIAA1549	102494167	102494700	4	533	-2.364824029	-5.871594539	hypo
CUX1	34727021	34727540	25	519	-1.15383668	-5.020222994	hypo
FMNL3	30201821	30202352	5	531	-1.564050898	-6.505171785	hypo
DNAJC22	30467870	30468157	5	287	-6.741466986	-5.059383236	hypo
ZC3HAV1	102708801	102709260	4	459	-1.172149232	-6.835831416	hypo
CLCA2	57619160	57619431	3	271	-6.855491443	-5.060220613	hypo
C18H16orf46	7745481	7746660	18	1179	-1.630801756	-5.090995289	hypo
TRANK1	10682961	10684036	22	1075	-4.06133106	-5.254546377	hypo
ZNF311	29995755	29996107	23	352	-2.435468727	-5.605562733	hypo

LHFPL4	17088303	17088792	22	489	-1.301083165	-5.025924741	hypo
TMEM106A	43175075	43176360	19	1285	-1.760829487	-5.081245653	hypo
ANKRD6	60560601	60562111	9	1510	-2.009640453	-5.232473829	hypo
SDC3	122701657	122702460	2	803	-5.590543533	-7.796532044	hypo
ABR	21860276	21860800	19	524	-1.144431113	-6.01163471	hypo
TRANK1	10681101	10681920	22	819	-3.750021747	-5.185577413	hypo
PIK3IP1	70095301	70096011	17	710	-2.379488078	-5.479845069	hypo
PCDHGA2	52490421	52491260	7	839	-1.803407978	-5.960235676	hypo
CD40	74852544	74853121	13	577	-2.152397835	-6.260971718	hypo
TUBB3	14715944	14716640	18	696	-1.594845197	-5.193277575	hypo
ZNF746	112486621	112488794	4	2173	-1.536377195	-5.616038335	hypo
ZAR1L	28684085	28685819	12	1734	-2.927886528	-5.253321993	hypo
DDX20	31540261	31541260	3	999	-1.330943283	-5.890631703	hypo
FAT2	62703103	62703561	7	458	-3.140611493	-6.121209708	hypo
CENPB	51419141	51419763	13	622	-2.619522084	-7.518317065	hypo
KAT5	43885842	43886391	29	549	-1.185435985	-6.042372504	hypo
EPS8L1	62312790	62313019	18	229	-1.033784946	-7.378111098	hypo
PCDHGA8	52474726	52475140	7	414	-4.554023791	-7.763595123	hypo
EPHX1	28870459	28870961	16	502	-2.229283252	-7.168791379	hypo
RALGAPA1	45843581	45844375	21	794	-1.293642123	-6.401106225	hypo
LZTS2	21841196	21842320	26	1124	-1.976481685	-7.267072583	hypo
PALM	43322315	43324385	7	2070	-1.487316856	-6.201014263	hypo
LIPT1	4312941	4313980	11	1039	-2.795962329	-6.007042869	hypo
ADAMTS14	26920556	26921580	28	1024	-1.287016142	-5.328122287	hypo
NFIX	12547513	12548045	7	532	-1.475796017	-6.693403959	hypo
MTFR1L	127239161	127239777	2	616	-1.512630635	-7.010627573	hypo
ACSS2	64232901	64233260	13	359	-2.249529618	-5.3884391	hypo
CCDC102A	25505115	25505325	18	210	-2.899589619	-5.012166142	hypo
MN1	67217885	67223040	17	5155	-4.65234543	-5.700954586	hypo
TEP1	26715161	26715680	10	519	-1.759249454	-7.201197757	hypo
LRFN4	44871336	44872695	29	1359	-1.142923584	-7.141250448	hypo
ZBTB33	4823681	4824000	30	319	-1.538867528	-5.258933973	hypo
RAB3D	15738681	15739080	7	399	-2.084839942	-7.228452597	hypo
PCLO	37457137	37458791	4	1654	-1.485844567	-5.59864345	hypo
ATP7B	21375510	21376560	12	1050	-1.129029253	-6.139759995	hypo
ANGPTL2	97839854	97840125	11	271	-1.482002689	-6.290542339	hypo
TBC1D30	48952181	48953213	5	1032	-1.259297107	-6.378113715	hypo
USP3	46413661	46413950	10	289	-1.478432581	-5.755140122	hypo
MEGF8	50921903	50922340	18	437	-2.040680617	-5.295858981	hypo
DAP3	15050241	15050920	3	679	-2.066758763	-5.880185829	hypo
CEP290	17879243	17879660	5	417	-1.256028673	-5.221511353	hypo
OSR1	79410776	79411475	11	699	-1.279723053	-6.351373543	hypo
PALMD	43540938	43541920	3	982	-3.292714739	-6.632105241	hypo
DTX3L	66993801	66994381	1	580	-3.813647766	-7.537522733	hypo
ZC3H4	54116580	54116829	18	249	-1.467545942	-5.238910118	hypo
LDB1	22779581	22780412	26	831	-1.036832355	-5.702229878	hypo
TMEM30B	73389981	73391840	10	1859	-1.167153374	-6.569182975	hypo
FASN	50794121	50794920	19	799	-1.655464372	-6.676478887	hypo
NIF3L1	89533181	89533868	2	687	-1.494586826	-6.924459751	hypo
PC	44863701	44863924	29	223	-1.733129995	-5.798368596	hypo
TTC3	149402332	149402700	1	368	-1.254712866	-7.094712238	hypo
FLYWCH1	2363721	2364420	25	699	-3.48521006	-7.691080923	hypo
SERTAD4	73095521	73095760	16	239	-1.96832754	-5.081751266	hypo
LOC786553	52346001	52346525	7	524	-2.85278319	-5.707387913	hypo
MAP10	5677641	5678420	28	779	-4.045610597	-6.239357504	hypo
QPCTL	53262235	53263008	18	773	-1.001134059	-5.525381579	hypo
ZC3H4	54119911	54120255	18	344	-1.270221129	-7.381914747	hypo
FDFT1	7588321	7589280	8	959	-1.793128084	-6.381981574	hypo

PBXIP1	15643041	15644005	3	964	-1.590752863	-6.302860859	hypo
YTHDF3	28171470	28171720	14	250	-1.016656219	-5.74320997	hypo
MOGS	10166481	10167320	11	839	-1.912601253	-5.406913247	hypo
KLHL9	22874269	22874740	8	471	-1.59489585	-7.118911638	hypo
FAT2	62725734	62726545	7	811	-3.002976062	-6.103501077	hypo
SIPA1L1	83237790	83238233	10	443	-1.132857392	-6.208310894	hypo
NACC2	103552754	103553698	11	944	-1.556913043	-7.185186606	hypo
TET1	25034841	25035820	28	979	-2.505458768	-5.104901272	hypo
ICOSLG	144223179	144223740	1	561	-1.448970294	-5.996281267	hypo
PRDM8	94886600	94888620	6	2020	-1.234471887	-7.259816571	hypo
LOC512672	28498603	28498879	23	276	-1.268553186	-5.150739673	hypo
CHST3	28097401	28098320	28	919	-1.410633561	-6.720455665	hypo
KIAA0895	60871861	60872456	4	595	-2.032677146	-5.216999037	hypo
PLCH2	50361621	50362360	16	739	-7.51412226	-6.25331942	hypo
PCDHGA2	52438421	52438920	7	499	-4.516249751	-5.729243284	hypo
NAB2	56343802	56344676	5	874	-2.105475976	-5.387127341	hypo
UCN	72336075	72336639	11	564	-2.625058382	-5.446594191	hypo
RASSF6	88621461	88621800	6	339	-8.613605318	-7.864766013	hypo
PCDHGA2	52596894	52597320	7	426	-1.762947983	-5.906326869	hypo
PCLO	37643037	37643796	4	759	-1.795482179	-5.405114796	hypo
GNPAT	4045309	4045613	28	304	-1.277438417	-6.253082275	hypo
KCTD14	18025501	18026233	29	732	-1.506634408	-5.059929714	hypo
LOC112442214	58376041	58376770	18	729	-1.659366749	-5.183550439	hypo
DIDO1	54507921	54508372	13	451	-1.370940039	-5.289865437	hypo
DDX58	11604221	11604700	8	479	-5.710218453	-6.188151604	hypo
TSPYL4	34447804	34449460	9	1656	-1.249120422	-6.591477955	hypo
SEMA6D	62640841	62642340	10	1499	-1.98981938	-7.532017812	hypo
SUPT7L	72032795	72033380	11	585	-1.595892061	-5.732618625	hypo
GGT6	25040645	25042088	19	1443	-3.041563811	-6.081363719	hypo
TP63	77498392	77498619	1	227	-1.757153481	-5.146424845	hypo
HOMEZ	21625944	21627680	10	1736	-2.205934451	-5.913371788	hypo
NCOA5	74798101	74798546	13	445	-1.761929938	-7.502291561	hypo
IL17RD	44002624	44003180	22	556	-1.477680315	-5.271945711	hypo
FOXN1	19991100	19991559	19	459	-3.674293392	-5.273007823	hypo
