# Supplementary material for: N6-Methyladenosine Modification Profile in Bovine Mammary Epithelial Cells Treated with Heat-Inactivated Staphylococcus aureus
Source: Oxid Med Cell Longev. 2022 Feb 23;2022:1704172. doi: 10.1155/2022/1704172 (PMC8890870; doi:10.1155/2022/1704172)
Supplement: Supplementary Materials — Table S1: differentially methylated RNA sites for S. aureus and con. Table S2: differential mRNA expression for S. aureus and Con. [file 1704172.f1.zip › Table S2.pdf]

Table S2. Differential mRNA expression for: *S. aureus* _vs_Con							
**Gene_id**	**logFC**	**P Value**	**Regulation**	**Gene_id**	**logFC**	**P Value**	**Regulation**
ADGRE3	6.486036	8.04E-05	up	FBN1	-8.47045	5.08E-08	down
STARD7	6.333711	0.000237	up	MYH11	-8.14671	5.39E-07	down
TRIP11	6.198409	7.93E-05	up	ACP2	-7.79738	4.6E-06	down
NTRK1	6.062762	0.000433	up	PRR29	-7.706764	9.69E-06	down
KRT80	6.019798	0.000639	up	LOC112444598	-7.592669	1.02E-05	down
LOC527796	6.005688	0.000884	up	CHRNB1	-7.551493	2.75E-05	down
RAB4B	5.879879	0.001578	up	PSIP1	-7.41187	0.00012	down
MAPKBP1	5.847415	0.001712	up	DXO	-7.372946	4.4E-05	down
PHOSPHO2	5.811408	0.001441	up	DSG1	-7.293155	0.000244	down
LOC100849008	5.810101	0.001884	up	GLS	-7.25391	0.000264	down
ARRDC1	5.781697	0.00255	up	CD70	-7.242189	0.000312	down
TBC1D24	5.781697	0.00255	up	BCKDHA	-7.209064	0.000191	down
RRM2	5.781697	0.00255	up	LOC509006	-7.172094	0.000358	down
CASS4	5.781697	0.00255	up	LOC112445660	-7.157447	0.000292	down
RNF170	5.652885	0.003358	up	LOC112444345	-7.050033	0.000444	down
DEPDC7	5.611438	0.003648	up	IFN-tau-c1	-7.038792	0.00033	down
CENPT	5.611438	0.003648	up	LHFPL4	-7.032866	0.000651	down
CNPY3	5.611438	0.003648	up	DDX39A	-7.027795	0.000708	down
GTF3C2	5.611438	0.003648	up	TMEM242	-7.027778	0.000542	down
TRMT13	5.611438	0.003648	up	INPP4A	-6.994488	0.000673	down
SLC2A1	5.579115	0.00066	up	KMT2E	-6.959519	0.000556	down
EXT1	5.563658	0.00251	up	PRPF38A	-6.94627	0.000947	down
STK40	5.559045	0.004067	up	TNK1	-6.929564	0.000843	down
RGS19	5.559045	0.004067	up	FAM186B	-6.895752	0.001088	down
CDC23	5.559045	0.004067	up	IFNT2	-6.876103	0.000921	down
ZNF446	5.522388	0.005451	up	PMEL	-6.855888	0.001024	down
POLR2K	5.489806	0.005755	up	CEACAM16	-6.855686	0.001157	down
ABCA4	5.379293	0.006882	up	CDAN1	-6.781825	0.001225	down
NTRK2	5.365955	3.79E-22	up	SPSB3	-6.767118	0.001325	down
PHYHD1	5.349989	0.005813	up	CMTM4	-6.763585	0.000721	down
SS18L1	5.348969	0.007238	up	MUTYH	-6.737135	0.001833	down
BMP1	5.347172	0.004792	up	LOC101906182	-6.710815	0.001397	down
DNAH5	5.313952	0.007653	up	ZBTB26	-6.701327	0.001986	down
RNF185	5.303605	0.007785	up	SLC12A5	-6.678912	0.002202	down
KLF13	5.213776	0.017491	up	IFNT3	-6.67316	0.00176	down
ADAMTS10	5.213776	0.017491	up	LRRC57	-6.636499	0.001529	down
LOC511695	5.213776	0.017491	up	PALLD	-6.627274	0.001462	down
ARHGEF10L	5.213776	0.017491	up	ARG2	-6.623666	0.002115	down
LIAS	5.213776	0.017491	up	CCDC146	-6.619334	0.002288	down
CENPE	5.213776	0.017491	up	KIF9	-6.569849	0.003153	down
CCDC171	5.213776	0.017491	up	FBXO24	-6.558497	0.002554	down
CCDC127	5.213776	0.017491	up	FAM210A	-6.557049	0.002665	down
KCTD5	5.196963	0.007539	up	SAP18	-6.555771	0.003062	down
ALK	5.186728	1.52E-07	up	CADM4	-6.554194	0.002456	down
LRCH1	5.158959	0.018354	up	KLHL7	-6.530291	0.003534	down
MRPS35	5.128358	0.018839	up	SLC26A1	-6.508133	0.003275	down
CEP55	5.103088	0.019264	up	SUPT7L	-6.501865	0.002675	down
DAB2	5.103088	0.019264	up	SLC25A51	-6.498936	0.003473	down
DNAJB6	5.103088	0.019264	up	LOC112442246	-6.493661	0.00391	down
MMP9	5.047204	0.006541	up	FNDC10	-6.487859	0.003132	down
MAP3K2	5.046789	0.006973	up	TUBGCP4	-6.451858	0.004216	down
MEAF6	5.046125	0.001813	up	OAZ2	-6.432691	0.003886	down
IRF2BP1	5.044854	0.009566	up	TAF10	-6.427388	0.003579	down
GPD2	5.043758	0.011652	up	GTPBP1	-6.423734	0.004269	down
MED22	5.043758	0.011652	up	CFB	-6.379163	0.004501	down

EYA3	5.040548	0.020297	up	SLC6A4	-6.349424	0.005716	down
EXOC3L4	5.040548	0.020297	up	SPACA9	-6.347493	0.003027	down
BHLHE23	5.040548	0.020297	up	KLK12	-6.318277	0.005765	down
GRIA4	5.040548	0.020297	up	ACTB	-6.303976	0.004408	down
PLCH1	5.040548	0.020297	up	IRF1	-6.291878	0.005966	down
ADAMTS1	5.040548	0.020297	up	RPF1	-6.285061	0.003283	down
NDUFC1	5.040548	0.020297	up	LYN	-6.272323	0.004847	down
MAFG	5.040548	0.020297	up	LOC112441554	-6.267936	0.005024	down
PALD1	5.040548	0.020297	up	ALKBH2	-6.264833	0.007034	down
C25H16orf92	5.040548	0.020297	up	LOC784768	-6.258719	0.007146	down
LOC507428	5.040548	0.020297	up	CIB1	-6.258094	0.005356	down
SERINC2	5.040548	0.020297	up	HBQ1	-6.252126	0.007269	down
C26H6orf52	5.040548	0.020297	up	LOC112442049	-6.247596	0.007308	down
RPGR	5.040548	0.020297	up	MANSC4	-6.242847	0.004919	down
ATG4C	5.040548	0.020297	up	GORASP1	-6.224529	0.005064	down
DDX41	5.040548	0.020297	up	AAGAB	-6.215815	0.005474	down
LOC617406	5.040548	0.020297	up	DSG4	-6.197694	0.007977	down
CIDEA	5.040548	0.020297	up	KRT5	-6.192795	0.007504	down
DEDD2	5.040548	0.020297	up	CACNB3	-6.19235	0.008328	down
PLA2G4E	5.040548	0.020297	up	COL18A1	-6.173006	0.006727	down
DCP1A	5.040548	0.020297	up	CCDC73	-6.166853	0.004907	down
ERO1A	5.021293	0.002801	up	LYL1	-6.146915	0.007865	down
ATRX	5.014538	0.020757	up	PRPF40A	-6.141438	0.008274	down
LOC112442307	4.997972	0.010355	up	ALOX12E	-6.135273	0.009497	down
WDR4	4.997764	0.010512	up	LHFPL2	-6.128203	0.009641	down
NR1D2	4.995067	0.002729	up	TBATA	-6.125312	0.008589	down
CYP19A1	4.988299	0.021228	up	TRAPPC13	-6.116893	0.008955	down
ARSI	4.988299	0.021228	up	JMJD4	-6.109483	0.010034	down
PRKDC	4.988299	0.021228	up	ARL13A	-6.107036	0.009785	down
MDFI	4.988299	0.021228	up	AHNAK2	-6.095125	0.006299	down
DCST1	4.988299	0.021228	up	CUL9	-6.080343	0.010074	down
LOC788915	4.988299	0.021228	up	DNAJC16	-6.066563	0.009088	down
C25H16orf91	4.988299	0.021228	up	HPCA	-6.066384	0.008332	down
MTMR12	4.988299	0.021228	up	NR2C2	-6.045211	0.011165	down
POLR1B	4.988299	0.021228	up	FXR1	-6.024283	0.01013	down
BROX	4.988299	0.021228	up	CLTA	-6.019532	0.008371	down
MPDU1	4.988299	0.021228	up	TANC1	-6.019119	0.012149	down
SNX31	4.988299	0.021228	up	B4GALNT4	-6.010008	0.012421	down
PRR15	4.988299	0.021228	up	PHB2	-5.997116	0.009923	down
CCDC68	4.988299	0.021228	up	LOC107132949	-5.996143	0.011346	down
SKA2	4.988299	0.021228	up	PAN2	-5.99249	0.012472	down
EVA1A	4.988299	0.021228	up	CDC42SE2	-5.991656	0.011336	down
ERCC4	4.988299	0.021228	up	ARSE	-5.991388	0.011101	down
H2AFV	4.988299	0.021228	up	TFR2	-5.984653	0.011298	down
ZNF432	4.988299	0.021228	up	TSR1	-5.983147	0.01236	down
MBNL3	4.988299	0.021228	up	VPS9D1	-5.975132	0.009221	down
ZC3H6	4.988299	0.021228	up	EEF1AKMT1	-5.966009	0.010989	down
NTRK3	4.871423	4.69E-16	up	NOTCH4	-5.963977	0.013198	down
SLC38A10	4.862384	0.003093	up	GNPDA1	-5.961314	0.01372	down
SMAD4	4.855829	4.01E-08	up	NT5C3A	-5.953043	0.009701	down
NOTCH2	4.841615	0.003374	up	RAB3GAP2	-5.945011	0.01326	down
ODF3	4.801236	0.00462	up	HEATR4	-5.941809	0.013024	down
C18H19orf47	4.801102	0.027541	up	LOC100336564	-5.940013	0.012741	down
PDP1	4.801102	0.027541	up	SPAM1	-5.939316	0.013884	down
FIBP	4.801102	0.027541	up	RCN3	-5.937783	0.009954	down
UPF3B	4.801102	0.027541	up	SLC44A3	-5.937316	0.011825	down
ECI1	4.7813	0.013269	up	RSPH9	-5.93424	0.012935	down
IL17RE	4.749079	0.028739	up	LNX1	-5.933697	0.010269	down

ROS1	4.733659	0.028159	up	LOC505972	-5.928049	0.014726	down
NARS	4.733659	0.029113	up	SMIM35	-5.928049	0.014726	down
TEAD4	4.733659	0.029113	up	FADS2	-5.927477	0.011738	down
NUP54	4.696377	0.029985	up	WNT2B	-5.927289	0.014782	down
MGA	4.694352	0.01947	up	SSR3	-5.914028	0.015211	down
CHEK2	4.680738	0.029377	up	ZNF274	-5.899382	0.015051	down
PRR13	4.680738	0.030374	up	FZD5	-5.892491	0.015284	down
LOC789503	4.679978	0.019568	up	TRMO	-5.883148	0.012388	down
PDGFA	4.665029	0.030768	up	CDC25B	-5.870054	0.015891	down
AGO3	4.665029	0.030768	up	ATG16L2	-5.869218	0.014334	down
SLC29A1	4.665029	0.030768	up	HTR7	-5.853145	0.014323	down
NFE2	4.665029	0.029754	up	RNF214	-5.85022	0.014491	down
ABLIM1	4.665029	0.030768	up	BHMG1	-5.848168	0.016156	down
CPLANE2	4.665029	0.029754	up	SENP8	-5.839481	0.014707	down
ACSS2	4.643042	0.031279	up	ZNF583	-5.834826	0.01447	down
LOC100139549	4.643042	0.030179	up	TULP1	-5.831711	0.015346	down
PCGF3	4.643042	0.031279	up	YBX3	-5.824521	0.015496	down
ADIPOR2	4.643042	0.031279	up	ZCCHC14	-5.809837	0.018031	down
IMPA2	4.643042	0.031279	up	LOC101906178	-5.808713	0.016903	down
PPP1R1B	4.639272	0.008754	up	DPYSL3	-5.808312	0.016124	down
PCYT1B	4.602276	0.021563	up	CDK13	-5.802076	0.015168	down
MXD1	4.602252	0.021595	up	VILL	-5.800472	0.019471	down
STRN	4.595311	0.032506	up	NDUFB9	-5.798769	0.017241	down
SPAG5	4.595311	0.032506	up	MXRA8	-5.798619	0.019494	down
CLUH	4.595311	0.032506	up	PRKAA1	-5.798486	0.019552	down
IARS	4.595311	0.031454	up	MASP1	-5.789587	0.019852	down
ZGLP1	4.595311	0.032506	up	AK5	-5.777021	0.020405	down
PSMD14	4.595311	0.032506	up	SYNPO2L	-5.775956	0.014004	down
PHF5A	4.520383	0.013921	up	LAMA5	-5.775225	0.016526	down
LOC100336029	4.451131	0.000192	up	GCHFR	-5.76975	0.017295	down
BCAM	4.413266	9.68E-05	up	SERPINE3	-5.768709	0.017275	down
SH3YL1	4.394295	0.014027	up	CDH23	-5.765799	0.012067	down
NRAS	4.342249	0.000279	up	ZBTB32	-5.764706	0.016532	down
NECTIN4	4.330383	0.005454	up	BAG2	-5.756848	0.020233	down
FGFR2	4.321818	0.001282	up	HACD3	-5.750181	0.019492	down
LARS2	4.316499	0.019671	up	MCOLN1	-5.739154	0.022031	down
MET	4.307432	0.000929	up	YARS	-5.732711	0.022309	down
HIST2H2BE	4.225383	0.047719	up	PC	-5.722043	0.016874	down
MRPL45	4.225383	0.047944	up	LOC100848495	-5.718523	0.013643	down
ATF6	4.225383	0.047951	up	TUBG1	-5.713824	0.019456	down
CYR61	4.225383	0.047839	up	CLCA3	-5.704737	0.023535	down
EPPK1	4.225383	0.047959	up	ANGPTL8	-5.695223	0.02182	down
FOS	4.225383	0.047959	up	PTPDC1	-5.685707	0.022042	down
GGCX	4.225383	0.047959	up	MARCKSL1	-5.67929	0.01604	down
GPBAR1	4.202645	0.02939	up	DLGAP5	-5.676746	0.024937	down
EPB41L5	4.162835	0.031193	up	NLRP5	-5.67369	0.023824	down
ETS1	4.159467	0.004912	up	CASKIN1	-5.665441	0.025473	down
CD19	4.158691	0.031325	up	HECTD1	-5.659437	0.019255	down
KCNH4	4.158691	0.031325	up	KIF20A	-5.655568	0.020775	down
CHD9	4.133166	0.022894	up	CHD3	-5.651269	0.022795	down
BRD7	4.121094	0.020451	up	GNB4	-5.649708	0.026163	down
RND3	4.096813	0.032386	up	CCDC38	-5.646746	0.0221	down
APMAP	4.096637	0.032826	up	SNED1	-5.637508	0.021388	down
COMMD3	4.095045	0.037185	up	DIAPH1	-5.636093	0.02441	down
OAZ1	4.091027	0.024603	up	FAR2	-5.63459	0.024469	down
MARCH6	4.077598	0.015081	up	CIT	-5.630041	0.027213	down
LOXL2	4.077314	0.038069	up	ERMAP	-5.616943	0.020201	down
RYBP	4.059206	0.039334	up	GPR151	-5.61065	0.021871	down

RDH11	4.059007	0.039636	up	PAPOLG	-5.602946	0.027257	down
CPEB3	4.057125	0.042686	up	ADAM1A	-5.600825	0.028867	down
PFDN1	4.056953	0.042984	up	PPOX	-5.596715	0.026257	down
ARHGAP5	4.03015	0.007765	up	UBE2R2	-5.594552	0.023939	down
MXRA7	4.018104	0.045728	up	PLXNC1	-5.585956	0.017729	down
WFDC13	4.018104	0.045728	up	MRPL9	-5.585006	0.026621	down
EBF3	4.018104	0.045728	up	TSTA3	-5.583518	0.022298	down
TRIB2	4.018104	0.045728	up	C8H9orf84	-5.582808	0.023333	down
APC	3.99119	2.08E-10	up	FTSJ3	-5.579502	0.02504	down
PEX12	3.984394	0.047322	up	LOC112444151	-5.574454	0.024829	down
RIPOR1	3.955977	0.048719	up	LOC104968627	-5.563639	0.024584	down
PTCH1	3.939518	1.57E-05	up	COMMD6	-5.556483	0.023486	down
PUS7L	3.934211	0.002064	up	EEF2	-5.556219	0.022807	down
PTEN	3.895454	5.64E-14	up	TRIM59	-5.551647	0.029775	down
RALY	3.869349	0.026066	up	PRAM1	-5.549768	0.027151	down
TFRC	3.853567	0.04797	up	E2F5	-5.54909	0.024328	down
RAPGEF1	3.826638	0.044366	up	XPA	-5.547582	0.027584	down
SERTAD2	3.805727	0.007758	up	SLC16A13	-5.546806	0.027685	down
EGLN3	3.782932	0.041294	up	AIFM2	-5.546015	0.024772	down
KRAS	3.76302	4.47E-10	up	SLC12A4	-5.543364	0.021791	down
SWAP70	3.762296	0.036958	up	PVR	-5.542591	0.029019	down
NKTR	3.738323	0.035218	up	TRAF4	-5.538316	0.032463	down
DDR2	3.690184	0.003958	up	PRPF40B	-5.53315	0.029221	down
FNBP1	3.653429	0.022984	up	SBNO2	-5.531701	0.024457	down
OPN1SW	3.648783	0.040389	up	ANGPT2	-5.530223	0.032728	down
MAP3K13	3.644869	0.02015	up	CCDC59	-5.527031	0.031267	down
PFKFB2	3.589209	0.002676	up	SRRM2	-5.523751	0.022016	down
PIK3CA	3.57095	1.1E-13	up	RPL3	-5.522189	0.023976	down
HSPA5	3.538431	0.005175	up	LOC522763	-5.514508	0.016561	down
AHCYL1	3.514501	0.034092	up	TMEM120B	-5.513575	0.032077	down
EWSR1	3.420888	1.73E-08	up	DIXDC1	-5.510637	0.034016	down
AKAP13	3.406917	0.038143	up	LOC789031	-5.509752	0.033972	down
ANLN	3.360978	0.001324	up	VTI1B	-5.503085	0.017219	down
NSMCE2	3.33523	0.033101	up	DONSON	-5.501007	0.03287	down
LOC516742	3.312287	0.049825	up	LOC112448260	-5.498524	0.029852	down
KDM4C	3.309631	0.006442	up	GDA	-5.493571	0.034903	down
TPBG	3.294017	0.046464	up	ARHGEF4	-5.493212	0.028063	down
ERBB4	3.247014	0.037866	up	SDR39U1	-5.492553	0.019491	down
RABEPK	3.217568	0.044108	up	RIPOR3	-5.491568	0.035117	down
VDAC2	3.200758	0.022815	up	CAMK1G	-5.482251	0.035662	down
LOC100847700	3.179099	0.033857	up	KIAA1958	-5.480269	0.035775	down
AJUBA	3.124397	0.042397	up	LDHC	-5.476554	0.028706	down
LOC520626	3.077536	0.018615	up	NMT2	-5.473809	0.036158	down
LOC614376	3.020755	1.33E-06	up	TWIST2	-5.472719	0.027316	down
HIF1A	2.987986	8.78E-06	up	DNAJB1	-5.467394	0.021282	down
NIPSNAP3A	2.969852	0.00658	up	TRIM27	-5.465046	0.031663	down
ANTXR2	2.951021	0.037858	up	PHLDA1	-5.464305	0.026335	down
VARS	2.923989	0.033431	up	EPHX1	-5.464178	0.025299	down
MTMR4	2.9104	0.039004	up	HDGF	-5.462854	0.031933	down
BMPR1A	2.905518	0.042063	up	ATP5F1C	-5.461869	0.035133	down
SMAD7	2.879724	0.049335	up	POLR1C	-5.460754	0.033453	down
HSPA1A	2.862707	4.9E-05	up	NACC1	-5.457044	0.035523	down
THOC6	2.800523	0.02494	up	LOC525426	-5.454272	0.03389	down
PDLIM5	2.774213	0.0084	up	PTRH2	-5.45219	0.037566	down
BRAF	2.761462	1.92E-06	up	ASPN	-5.447869	0.035869	down
ZSWIM4	2.667581	0.005273	up	FSCN1	-5.442221	0.032959	down
SEPT1	2.661467	0.046151	up	RPS24	-5.441692	0.027748	down
EFCAB9	2.639314	0.008034	up	IL33	-5.440312	0.034804	down

CDK4	2.546783	0.005473	up	HHLA2	-5.438637	0.034713	down
TAF6L	2.508712	0.004843	up	EHF	-5.435032	0.036945	down
ERN2	2.474471	0.027779	up	UBP1	-5.432421	0.033704	down
UBXN1	2.368855	0.047542	up	B3GAT2	-5.431049	0.024265	down
TOR1AIP2	2.286356	0.008536	up	OGFOD1	-5.422408	0.037407	down
ST3GAL1	2.231874	0.014371	up	SYT5	-5.421992	0.030602	down
LOC529277	2.224482	0.026485	up	AUNIP	-5.418577	0.039778	down
ABCF1	2.192168	0.038941	up	ENO4	-5.416565	0.039781	down
ACTRT3	2.189265	0.043764	up	EXOSC3	-5.413882	0.032824	down
TOR1AIP1	2.170938	0.033461	up	WDR19	-5.410079	0.040315	down
PLCXD2	2.055349	0.009469	up	RCBTB1	-5.399182	0.039104	down
CCND1	1.987403	0.032328	up	KLF17	-5.39371	0.039252	down
TAF9B	1.915799	4.98E-05	up	COPG2	-5.393564	9.99E-12	down
SERPINE1	1.895457	0.01561	up	WDR93	-5.389971	0.041617	down
PPP1R11	1.889464	0.025211	up	DNAJC17	-5.389391	0.04153	down
SPP1	1.888299	1.16E-06	up	MCCD1	-5.389259	0.039641	down
TMSB10	1.28622	0.016707	up	AOX2	-5.386805	0.041951	down
STAG3	1.226201	0.00502	up	COL7A1	-5.386696	0.023603	down
LOC112445011	1.074134	1.05E-05	up	ADCY7	-5.380803	0.039943	down
LOC101904601	1.051453	0.025978	up	TMEM80	-5.380024	0.034052	down
				EPGN	-5.377571	0.035492	down
				MPPE1	-5.370856	0.03917	down
				LOC616819	-5.361954	0.028416	down
				SMC4	-5.360249	0.041798	down
				CRIM1	-5.354811	0.04393	down
				CASZ1	-5.351544	0.044285	down
				ITGB3BP	-5.345507	0.044691	down
				CYB5R2	-5.341626	0.044957	down
				SCYL3	-5.339196	0.031121	down
				ACIN1	-5.338826	0.034078	down
				ADAM1B	-5.337836	0.045078	down
				LOC518106	-5.333406	0.045668	down
				TAGLN2	-5.331452	0.03059	down
				ATP1B1	-5.330717	0.045566	down
				ANKRD53	-5.329784	0.04156	down
				MMP13	-5.329678	0.021342	down
				NANOS1	-5.328068	0.045887	down
				DUSP16	-5.32474	0.046262	down
				FBXW4	-5.323181	0.044234	down
				UGP2	-5.313312	0.044624	down
				SETD6	-5.311114	0.041013	down
				NCAPD2	-5.310313	0.026823	down
				LOC112441471	-5.306449	0.0474	down
				TRIP12	-5.306343	0.047558	down
				NECAP2	-5.303407	0.047764	down
				CSTB	-5.303091	0.034604	down
				DSG3	-5.302789	0.045251	down
				TNFSF14	-5.301964	0.045656	down
				LIG3	-5.30154	0.045835	down
				MFSD4A	-5.295194	0.045769	down
				TMEM243	-5.294095	0.037638	down
				SRPRA	-5.293154	0.048494	down
				MAPKAPK5	-5.29168	0.046129	down
				AFF1	-5.286529	0.042497	down
				DAZAP2	-5.285536	0.039314	down
				PRR15L	-5.284452	0.044756	down
				ASAP1	-5.283859	4.13E-07	down
				FTL	-5.280306	0.042596	down

				SPG11	-5.278522	0.03216	down
				GPM6B	-5.278422	0.04956	down
				ITPR3	-5.275609	0.047248	down
				SLC35G2	-5.271904	0.047741	down
				KLHL36	-5.270468	0.047832	down
				STX19	-5.262761	0.033934	down
				SMPDL3B	-5.259235	0.036877	down
				ANKRD33B	-5.254511	0.031795	down
				NT5E	-5.252837	0.04899	down
				TMUB2	-5.245016	0.0408	down
				ASH1L	-5.241972	0.047854	down
				GOLGA2	-5.232368	0.037324	down
				FBXO17	-5.229025	0.048886	down
				F3	-5.226483	0.041575	down
				CTNND1	-5.198415	0.043603	down
				ISYNA1	-5.189294	0.047631	down
				MIS18BP1	-5.16228	0.049518	down
				BRI3	-5.160254	0.039596	down
				TMEM200B	-5.159388	4.23E-07	down
				TSC22D1	-5.129515	0.039471	down
				TNFRSF6B	-5.122925	0.048857	down
				RPL13	-5.108571	0.043749	down
				PLEKHG3	-5.1082	0.045854	down
				TRMT112	-5.101255	0.049973	down
				ABRAXAS1	-5.097186	0.044833	down
				SLC50A1	-5.090369	0.049109	down
				MAPK8IP1	-5.037099	0.048966	down
				GLDC	-4.828479	8.26E-08	down
				LOC101908039	-4.810298	6.7E-08	down
				PPP2R1B	-4.71382	2.47E-10	down
				ARMC10	-4.449183	0.000148	down
				PKD1L2	-4.432874	2.89E-06	down
				CCNE2	-4.334496	0.000406	down
				SDCCAG8	-4.329093	3.47E-06	down
				MLLT11	-4.300492	0.000642	down
				PDE8B	-4.254643	0.000654	down
				SCYL1	-4.253178	4.74E-06	down
				EIF3B	-4.216985	0.000457	down
				CCDC66	-4.199432	2.79E-08	down
				FANCL	-4.018752	4.39E-05	down
				C2H2orf88	-3.871844	8.51E-05	down
				ACOT8	-3.851475	0.003915	down
				ZAR1L	-3.798677	0.00036	down
				HOXA10	-3.787842	0.005559	down
				YEATS2	-3.787444	0.006704	down
				PPEF2	-3.778651	0.000142	down
				APBA3	-3.767055	0.003096	down
				RASGRP1	-3.753849	0.007391	down
				RBM4B	-3.75252	0.000266	down
				PLCL2	-3.725879	0.000306	down
				CLIC3	-3.711025	5.84E-06	down
				C7H19orf44	-3.700024	0.000391	down
				ZBTB37	-3.693594	0.000561	down
				REC114	-3.65419	0.006827	down
				SMPD3	-3.626839	0.009822	down
				CD160	-3.623793	0.010466	down
				TMEM140	-3.616055	0.008454	down
				ADGRF2	-3.53764	0.012825	down

				NSG1	-3.527522	0.000593	down
				ADAL	-3.525676	0.001191	down
				EMG1	-3.511732	0.01015	down
				LOC532875	-3.50534	7.39E-06	down
				STK32A	-3.504329	0.014856	down
				AIF1	-3.500181	0.011815	down
				BBIP1	-3.49996	1.25E-06	down
				KLK11	-3.497294	0.001849	down
				NIT1	-3.465559	0.013608	down
				TOPORS	-3.4547	0.010231	down
				C15H11orf49	-3.452809	0.015717	down
				TRIM13	-3.431502	0.000424	down
				SLK	-3.427056	0.013133	down
				DNAJC24	-3.415661	3.03E-09	down
				NEPRO	-3.408989	0.000286	down
				LOC112442013	-3.408796	0.020468	down
				YIF1B	-3.39513	0.000222	down
				DNASE1	-3.386731	0.018037	down
				DDB2	-3.372103	1.15E-06	down
				CACNB2	-3.369884	0.02008	down
				MAGI3	-3.35978	0.00046	down
				NUP88	-3.346504	0.018247	down
				TCP1	-3.33055	4.65E-07	down
				BUD31	-3.314974	0.023241	down
				CDK17	-3.30666	0.018376	down
				PPHLN1	-3.292927	0.022798	down
				ABCA7	-3.29024	0.024675	down
				PDZD2	-3.288496	0.018	down
				SLC41A3	-3.284577	0.024396	down
				AKAP5	-3.27064	0.002678	down
				ACTA2	-3.253269	0.002114	down
				DDX49	-3.248155	0.020011	down
				IFITM5	-3.228552	0.029282	down
				HEXIM1	-3.21646	0.032186	down
				LOC100847454	-3.207403	1.12E-05	down
				STRBP	-3.196971	1.67E-06	down
				DCUN1D3	-3.193261	0.032234	down
				C5H12orf56	-3.191997	0.032359	down
				MYO1H	-3.191342	0.005943	down
				PIGB	-3.185363	0.004392	down
				MXI1	-3.174716	0.034451	down
				GPR183	-3.167478	0.03479	down
				OR9Q2	-3.157636	0.035937	down
				LOC112448894	-3.14536	0.028156	down
				STAT4	-3.139937	0.035486	down
				LOC107131992	-3.12593	0.035786	down
				GAS8	-3.121172	0.000672	down
				NSUN7	-3.107024	0.041361	down
				CLU	-3.074323	0.043349	down
				ZNF677	-3.053609	0.006083	down
				FOXRED2	-3.043761	0.040911	down
				GPN1	-3.041142	0.048923	down
				MRPL55	-3.03585	0.038028	down
				PCNT	-3.031809	0.04848	down
				EIF2S1	-3.031098	0.011088	down
				TRIM17	-3.019139	0.045337	down
				LRRC39	-3.017463	0.005223	down
				PASK	-3.014531	0.048764	down

				CRB2	-3.005744	0.032618	down
				GUF1	-3.00506	0.002229	down
				SMIM33	-2.973818	0.01288	down
				VGF	-2.942097	0.000659	down
				ATXN7L3	-2.915032	0.010308	down
				GNPNAT1	-2.911963	0.001412	down
				TRNT1	-2.897265	0.013057	down
				RPE	-2.894847	0.016729	down
				HGFAC	-2.894607	0.017434	down
				NSD1	-2.890195	0.048209	down
				MOG	-2.887699	2.6E-11	down
				LRRC19	-2.864338	0.009967	down
				PP2D1	-2.864222	0.000156	down
				CFAP126	-2.846078	0.049525	down
				EID1	-2.836352	0.047617	down
				RNF227	-2.831081	9.95E-06	down
				GPATCH11	-2.827985	0.004043	down
				LOC782781	-2.827407	0.019417	down
				GGACT	-2.769863	0.021094	down
				LOC104974020	-2.744888	0.022784	down
				CCDC189	-2.739341	0.000489	down
				PLTP	-2.73677	0.006883	down
				POLR2B	-2.691258	0.000161	down
				CENPBD1	-2.679383	0.001295	down
				CDKN2B	-2.676771	0.000363	down
				ZRANB1	-2.663339	0.024708	down
				PAF1	-2.658896	0.025373	down
				KRR1	-2.645538	0.033133	down
				AGBL3	-2.631356	0.008779	down
				CEACAM1	-2.620234	0.011564	down
				TUBA1C	-2.610969	6.95E-07	down
				WDR72	-2.594474	0.003216	down
				MEGF11	-2.592279	5.89E-05	down
				SLC16A4	-2.585895	2.32E-06	down
				TRIM52	-2.572418	0.000199	down
				SH2B3	-2.551483	0.000153	down
				KCNRG	-2.542766	0.021005	down
				AQP11	-2.540755	0.047084	down
				OMG	-2.537765	2.99E-05	down
				RAB24	-2.537446	0.007273	down
				GAPDHS	-2.531448	0.048632	down
				ABCC3	-2.530229	0.046255	down
				FER1L5	-2.51938	0.003894	down
				SLC23A1	-2.487349	0.048262	down
				KRI1	-2.484304	0.032735	down
				SPTB	-2.48392	1.39E-07	down
				CCAR2	-2.481498	0.048047	down
				MAPK15	-2.479728	0.000833	down
				CC2D2A	-2.465854	0.039225	down
				HMGA1	-2.457727	0.043166	down
				FBXL14	-2.457259	1.21E-07	down
				NR2C2AP	-2.444656	0.004084	down
				LY6G6F	-2.438668	2.69E-05	down
				LOC112448863	-2.438571	0.045391	down
				AKAP9	-2.43449	0.036544	down
				BAZ2A	-2.419758	0.022781	down
				LOC101906018	-2.409485	0.000673	down
				LOC107132617	-2.40296	0.020939	down

				CD3EAP	-2.399614	0.020369	down
				TMEM175	-2.395657	0.031096	down
				PAICS	-2.356282	0.01542	down
				GREB1L	-2.347917	0.00096	down
				LARGE2	-2.340148	0.000108	down
				FANCI	-2.321105	0.01206	down
				WDR6	-2.310326	0.033931	down
				ORAOV1	-2.306729	0.011682	down
				LYPD1	-2.268171	0.002384	down
				FRY	-2.266885	0.000172	down
				TTC28	-2.250212	0.002211	down
				FUZ	-2.24976	0.000269	down
				ZNHIT3	-2.246333	0.003807	down
				EVI2A	-2.244341	0.003618	down
				ZBTB11	-2.233872	0.044514	down
				C16H1orf112	-2.220361	0.001691	down
				DENND1C	-2.20245	6.4E-06	down
				PHYKPL	-2.193952	0.030888	down
				BMP6	-2.182163	0.011492	down
				COL17A1	-2.174146	0.039968	down
				CBARP	-2.172614	0.02709	down
				PDCD11	-2.170702	0.031571	down
				FBXL20	-2.156806	4.62E-07	down
				LOC509034	-2.155765	2.47E-09	down
				NDUFAF7	-2.144957	0.001003	down
				MZF1	-2.143258	0.020112	down
				HERC5	-2.135917	0.017498	down
				LOC107132994	-2.135894	0.001058	down
				TMOD1	-2.130997	0.005129	down
				LOC104975027	-2.129607	0.030803	down
				FASN	-2.124688	0.039568	down
				RFXANK	-2.118634	0.03128	down
				C15H11orf42	-2.11349	0.027941	down
				TM6SF1	-2.112373	0.001534	down
				LOC112447324	-2.110051	0.000124	down
				LENG9	-2.108549	0.001669	down
				ZAN	-2.089297	0.00921	down
				MAK16	-2.087308	0.045162	down
				CSTF3	-2.072308	0.047506	down
				ZC3H14	-2.043603	0.024208	down
				CHRNA2	-2.043159	0.005731	down
				NAPEPLD	-2.010057	3.37E-05	down
				TIAM2	-2.000896	0.035266	down
				LOC100848357	-1.980077	0.003826	down
				SLC3A1	-1.958985	0.03759	down
				EVI2B	-1.95116	0.001528	down
				LOC112447762	-1.950179	1.36E-05	down
				LOC101902106	-1.946839	0.046295	down
				CCDC84	-1.939719	0.01412	down
				KDM7A	-1.931791	0.033599	down
				PRKAR1A	-1.925284	0.049168	down
				HSPA4L	-1.921369	0.048856	down
				LOC101905992	-1.91263	0.033136	down
				LIPM	-1.906762	0.024252	down
				ERMP1	-1.86666	0.026074	down
				ZNF729	-1.862714	0.04108	down
				LTBP3	-1.856159	0.00021	down
				SLC18A2	-1.85005	0.034981	down

				RAD54L	-1.844874	0.049101	down
				CSPG4	-1.840681	0.005271	down
				MRPL47	-1.839902	0.024053	down
				GFY	-1.838172	0.048088	down
				ZRANB3	-1.838054	0.044206	down
				THOC1	-1.835124	8.52E-07	down
				GNE	-1.801905	0.043125	down
				ESRP2	-1.792293	0.020666	down
				VWA2	-1.789262	0.024263	down
				TMEM216	-1.787144	2.81E-05	down
				ANKRD42	-1.783645	0.002354	down
				UPB1	-1.779742	0.012019	down
				TMCO6	-1.76382	0.00349	down
				TFIP11	-1.763074	0.039512	down
				NDUFA6	-1.745411	0.007341	down
				RPP38	-1.703362	0.012055	down
				MRNIP	-1.699397	0.007578	down
				PPWD1	-1.690602	0.04871	down
				TMEM45B	-1.67783	0.003398	down
				PRELID3A	-1.673887	0.000269	down
				NR1I3	-1.671587	0.016803	down
				VPS54	-1.667442	0.000731	down
				PIK3CD	-1.6621	9.06E-06	down
				FAM184B	-1.652952	4.74E-09	down
				GRTP1	-1.651903	0.000922	down
				MPHOSPH8	-1.646847	0.023049	down
				NAT9	-1.634668	0.023107	down
				DALRD3	-1.633139	0.030015	down
				LDLRAD2	-1.631572	0.013027	down
				PKMYT1	-1.626365	0.022275	down
				KDELR3	-1.621218	0.015741	down
				RNF113A	-1.601321	0.002354	down
				LRRC4	-1.58953	0.009332	down
				PHTF1	-1.585606	0.007585	down
				LOC112445988	-1.539353	0.048441	down
				YIPF2	-1.539201	0.005617	down
				CTNS	-1.525529	0.004074	down
				CD37	-1.517309	0.016211	down
				KLHL8	-1.506616	6.87E-07	down
				FAM161B	-1.504323	0.001747	down
				KCNJ13	-1.501692	0.004248	down
				DNAH7	-1.48691	0.009177	down
				LOC100848188	-1.481323	0.008678	down
				SHLD2	-1.475261	0.001191	down
				BBOF1	-1.440987	0.021481	down
				RAD9A	-1.428973	0.003085	down
				AGBL5	-1.423842	0.003242	down
				ZFYVE9	-1.407942	0.000185	down
				LOC112443225	-1.400785	0.001357	down
				LOC107132958	-1.383638	0.004171	down
				GPS2	-1.381872	2.44E-05	down
				GLRX2	-1.377349	0.049593	down
				ST7L	-1.372824	0.000569	down
				MTHFD2L	-1.360401	0.040867	down
				FN1	-1.350292	0.000167	down
				MAP3K15	-1.345281	0.003433	down
				RPS6KL1	-1.320671	0.004354	down
				NDEL1	-1.288969	0.007578	down

				MASP2	-1.27023	0.015536	down
				ATG7	-1.261962	0.015763	down
				PLPP1	-1.242423	0.011883	down
				LOC112448540	-1.237365	0.044528	down
				MIA3	-1.226954	2.46E-05	down
				KANSL1L	-1.207557	0.008976	down
				SURF2	-1.202532	0.001136	down
				BACE1	-1.190975	0.004687	down
				SLA2	-1.15939	0.014698	down
				ABHD12B	-1.152729	0.034079	down
				JMJD8	-1.141944	0.017318	down
				DIMT1	-1.141429	0.044346	down
				MAP6D1	-1.138598	0.013997	down
				EML6	-1.117696	0.002443	down
				METTL23	-1.105629	0.008592	down
				SLC24A5	-1.094819	0.039641	down
				GNAT2	-1.091777	0.005534	down
				MCC	-1.088441	0.046625	down
				SNF8	-1.082818	0.018843	down
				METTL5	-1.022838	0.042161	down
				TINF2	-1.004447	0.029016	down
				NEMP2	-1.00103	0.012814	down
